# Meeting report: Considerations for trial design and endpoints in licensing therapeutic HPV16/18 vaccines to prevent cervical cancer

**DOI:** 10.1016/j.vaccine.2024.07.001

**Published:** 2024-11-14

**Authors:** Peter M. Dull, Sharon L. Achilles, Rafi Ahmed, Ruanne V. Barnabas, Nicole G. Campos, Keith Chirgwin, Jamie A. Cohen, Silvia de Sanjosé, John Doorbar, Mark H. Einstein, Claudia I. Emerson, Sami L. Gottlieb, Allan Hildesheim, Youlin Qiao, Paul Ruff, Joshua N. Sampson, Peter Sasieni, Mark Schiffman, Haina Shin, Margaret A. Stanley, Cornelia L. Trimble, Nicholas Wentzensen, Angelika B. Riemer, John T. Schiller, Aimée R. Kreimer

**Affiliations:** a Bill & Melinda Gates Foundation, Seattle, WA, USA; b Emory Vaccine Center, Atlanta, GA, USA; c Division of Infectious Diseases, Department of Medicine, Massachusetts General Hospital, Boston, MA, USA; d School of Medicine, Harvard Medical School, Boston, MA, USA; e Harvard T.H. Chan School of Public Health, Boston, MA, USA; f Independent Consultant, Seattle, WA, USA; g ISGlobal, Barcelona, Spain; h National Cancer Institute, Bethesda, MD, USA; i University of Cambridge, Cambridge, UK; j Rutgers New Jersey Medical School, Newark, NJ, USA; k McMaster University, Hamilton, Ontario, Canada; l World Health Organization, Geneva, Switzerland; m Independent Consultant, Willis, VA, USA; n Chinese Academy of Medical Sciences and Peking Union Medical College, Beijing, China; o University of Witwatersrand Faculty of Health Sciences, Johannesburg, South Africa; p Queen Mary University of London, London, UK; q Division of Cancer Epidemiology and Genetics, National Cancer Institute, Bethesda, MD, USA; r Johns Hopkins University School of Medicine, Baltimore, MD, USA; s German Cancer Research Center (DKFZ), Heidelberg, Germany; t German Center for Infection Research (DZIF), Partner Site Heidelberg, Germany; u Center for Cancer Research, National Cancer Institute, Bethesda, MD, USA

**Keywords:** Cervical cancer, Human papillomavirus, HPV, HPV therapeutic vaccine

## Abstract

Cervical cancer is a major cause of morbidity and mortality globally with a disproportionate impact on women in low- and middle-income countries. In 2021, the World Health Organization (WHO) called for increased vaccination, screening, and treatment to eliminate cervical cancer. However, even with widespread rollout of human papillomavirus (HPV) prophylactic vaccines, millions of women who previously acquired HPV infections will remain at risk for progression to cancer for decades to come. The development and licensing of an affordable, accessible therapeutic HPV vaccine, designed to clear or control carcinogenic HPV and/or to induce regression precancer could significantly contribute to the elimination efforts, particularly benefiting those who missed out on the prophylactic vaccine. One barrier to development of such vaccines is clarity around the regulatory pathway for licensure.

In Washington, D.C. on September 12–13, 2023, a meeting was convened to provide input and guidance on trial design with associated ethical and regulatory considerations. This report summarizes the discussion and conclusions from the meeting. Expert presentation topics included the current state of research, potential regulatory challenges, WHO preferred product characteristics, modeling results of impact of vaccine implementation, epidemiology and natural history of HPV infection, immune responses related to viral clearance and/or precancer regression including potential biomarkers, and ethical considerations. Panel discussions were held to explore specific trial design recommendations to support the licensure process for two vaccine indications: (1) treatment of prevalent HPV infection or (2) treatment of cervical precancers. Discussion covered inclusion/exclusion criteria, study endpoints, sample size and power, safety, study length, and additional data needed, which are reported here. Further research of HPV natural history is needed to address identified gaps in regulatory guidance, especially for therapeutic vaccines intended to treat existing HPV infections.

## Highlights

• Effective HPV therapeutic vaccines could reduce HPV-related cancer and associated deaths.
• An expert convening was held in September 2023 to provide guidance on vaccine trial design.
• Study design for vaccines targeting cervical precancers has regulatory precedent.
• Licensure pathways for treating existing HPV infection is less clear.
• Early engagement with regulators with supportive data is strongly encouraged.

## 1 Introduction

Cervical cancer is the fourth most common cancer in women worldwide, with nearly 350,000 deaths in 2022 [1], [2]. The two main prevention methods, prophylactic human papilloma virus (HPV) vaccination and screening, early detection, and treatment of precancerous lesions, are less widely available in many low- and middle-income countries (LMICs), and these countries collectively bear a disproportionate burden (90 %) of cervical cancer deaths [3]. In 2021, the World Health Organization (WHO) called for a global initiative to eliminate cervical cancer through increased vaccination, screening, early detection and treatment of existing premalignant and malignant lesions [4].

The development of prophylactic HPV vaccines was a tremendous scientific achievement. Already, reductions in cervical cancer have been observed in high income countries, but enabling access to these vaccines worldwide remains challenging [5]. Progress has been made in introductions, but even with optimistic targets for scale-up, there will be decades where women already HPV-infected remain at risk for cervical cancer. Screening for cervical cancer, and treatment of identified precancers, remains challenging in many countries with the highest burden of cervical cancer. In these settings in particular, approaches such as anti-viral drugs (outside the scope of this report) and therapeutic HPV vaccines could play an important role in preventing cervical cancer.

There are two potential indications for a therapeutic HPV vaccine: treatment of HPV infection or treatment of cervical precancer. Although multiple products are in development, licensure will depend on successful late-phase clinical trials to demonstrate safety and efficacy. In September 2023, a group of epidemiologists, clinicians, researchers, regulators, product developers and other HPV experts convened to provide guidance on considerations for such studies. This report reviews the content of that meeting which included a review of the current state of the science and considerations of how to support continued therapeutic HPV vaccine development, with the aim to facilitate product developers in designing programs and provide guidance for regulators and/or ethics committees evaluating such programs. The sections attributed to individual authors summarize their own views on the topic and not necessarily the consensus opinion of the attendees.

### 1.1 Background and current landscape

Understanding the potential public health impact of a therapeutic HPV vaccine is essential for guiding product development and trial design. On the first day of the convening, experts reviewed the current state of HPV vaccine research including lessons from pivotal trials for prophylactic HPV vaccines, assessed the potential impact of therapeutic HPV vaccine implementation, and highlighted critical regulatory and design considerations for late-phase trials and licensure. Summaries of these sessions are included in the supplementary material.

#### 1.1.1 Regulatory perspective: Link between indication and clinical data requirements and use of surrogate endpoints to support labeling

After the introductory overviews, Keith Chirgwin reviewed regulatory concepts on the approach to seeking labeling claims. Efficacy claims are supported by clinical outcomes or by surrogate endpoints for clinical outcomes which must be well-defined, reliable and demonstrate a clinically meaningful effect.

Surrogate endpoints for clinical outcomes should be biologically plausible and have a known relationship with the clinical outcome. They should have demonstrated association with the clinical outcome, the association strength should be well established, and the association should show consistency across different populations and treatment regimens. Surrogate endpoints should demonstrate predictive value in clinical trials, i.e. the impact of an intervention on the surrogate should accurately predict the impact on the clinical endpoint of interest.

Given these requirements, assessing efficacy endpoints for HPV therapeutic vaccine trials may pose challenges. While prevention of cervical precancer is a widely accepted surrogate for prevention of cervical cancer, precancer regression as an endpoint relies on biopsy procedures which can alter the natural history of the lesion, thus confounding the assessment. Clearance of HPV infection (as indicated by a negative HPV test) is a biologically plausible potential surrogate endpoint for precancer prevention, but regulators may require additional data to support it as a valid surrogate endpoint. Sponsors should justify the methods used to define viral clearance including specimen collection, timepoints for assessment, duration of follow-up, and assay performance characteristics.

#### 1.1.2 Additional presentations related to current therapeutic HPV vaccine landscape

While there are important lessons from the licensure of prophylactic HPV vaccines (presented by John Schiller: Supplement Section 1), therapeutic HPV vaccine development has differences which limit applying the previous lessons learned. Sharon Achilles and Sami Gottlieb provided background on the potential public health need for therapeutic HPV vaccines and presented the WHO preferred product characteristics (PPCs) for therapeutic vaccines [6] which target cervical HPV infection and HPV-associated cervical precancer (Table 1 and Supplement Section 2). Haina Shin provided an overview of existing or past efforts at development for therapeutic vaccine products that target HPV, emphasizing the limited number of therapeutic HPV vaccines which have progressed to an advanced clinical development stage requiring detailed discussions with regulators (Supplement Section 3). Jamie Cohen summarized past results and presented new work from modeling studies describing how therapeutic HPV vaccines may contribute to accelerated and sustained long-term cervical cancer burden reduction, particularly if they provide action against high-grade lesions and durable immune memory (Supplement Section 4).

**Table 1:** WHO preferred product characteristics (PPCs) for therapeutic HPV vaccines.

*1a. Indication for PPC 1: Therapeutic HPV vaccines used to clear carcinogenic HPV infections.*	
PPC	Relevant notes
For first-generation vaccines: Clearance of carcinogenic HPV infection, at a minimum types 16 and 18 and/or prevention of high-grade cervical precancers associated with these HPV types	The goal of clearing carcinogenic infection would be preventing progression to high-grade cervical precancers, which in turn would be expected to prevent progression to cervical cancer.
Increased global public health value would result from additional vaccine activity in: Regression of cervical precancers AND/OR Clearance of additional carcinogenic HPV type infections, AND/OR Prolonged effects against reinfection or recurrences	Regulatory guidance will be needed to confirm whether durable clearance of infection as measured in clinical trials is an acceptable surrogate for prevention of cervical cancer. Discussions with regulators can also establish the appropriate time frame for measuring clearance and whether prevention of high-grade precancers should be evaluated instead of, or in addition to, clearance of infection in clinical trials. Efficacy in regressing precancers, cross-protection, and immune memory would expand public health benefits and could affect recommendations for broader use.

*1b. Indication for PPC 2: Therapeutic HPV vaccines used to treat cervical precancer.*	
PPC	Relevant notes
Regression of high-grade cervical precancers (i.e., CIN2/3), at a minimum those associated with HPV types 16 and 18 Regression of high-grade cervical precancers due to other carcinogenic HPV types or clearance of additional HPV infections or low-grade cervical lesions would have added benefit	Therapeutic HPV vaccines that regress cervical precancers and are preferable to existing treatments with respect to efficacy, safety, cost, delivery, and/or acceptability to women could be useful interventions
	They might also provide benefit as an adjunct to existing treatments in improving efficacy or reducing recurrences
	Clinical endpoints will need to be refined in discussion with regulators, including the time frame for assessing precancer regression and whether associated viral clearance is an essential component of the primary outcome and how to assess it

CIN, cervical intraepithelial neoplasia; HPV, human papilloma virus.

### 1.2 Epidemiology and natural history of HPV infection leading to cervical precancer

**Fig. 1:**
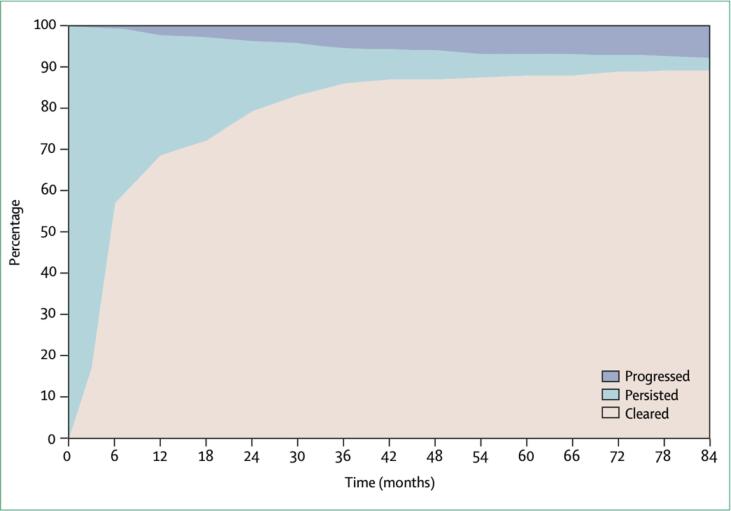
**Average clearance, persistence, and progression of carcinogenic HPV infections.** Carcinogenic HPV infections detected by DNA testing tend to resolve within one to two years of detection. Details vary by population, HPV type, and age, but this diagram of 777 infections found at enrolment visits of a large population-based cohort study (Guanacaste, Costa Rica) illustrates a typical pattern. Over time, the risk of a precancer diagnosis rises while the probability of eventual clearance among the still-persistent infections falls. This figure was published in The Lancet, Vol 370, Schiffman M, Castle PE, Jeronimo J, Rodriguez AC, Wacholder S, “Human papilloma virus and cervical cancer,” pp. 890–907, Copyright Elsevier. (2007).

Silvia de Sanjosé outlined the burden of carcinogenic HPV types and disease (Supplement Section 5) and Mark Schiffman and Nicole Campos described points of importance during the natural history of infection for evaluating therapeutic vaccines. HPV 16 and 18 are the most important carcinogenic types worldwide and are responsible for approximately 70 % of cervical cancers. Carcinogenic HPV infections that persist over time increase the risk of cervical precancer. The natural history of HPV infection and the causal pathway to invasive cervical cancer include necessary health states (type-specific HPV infection, precancer, cancer) with distinct transition risks between each of them (Fig. 1). An incident infection (i.e., new detection of an infection following negative test results) can represent a newly acquired infection, reactivation of a latent infection, or potential virus deposition from an infected partner, while transitioning from a detectable type-specific infection to non-detection can indicate elimination of the virus or latency. Progression to precancer occurs when a productive high-risk HPV infection becomes an abortive transforming infection generating a clone of cells with severe disruption of cellular growth and differentiation controls, decreased programmed cell death, and increased genetic instability. Importantly, precancer is not synonymous with histologic diagnoses of “CIN2” or “CIN2/3”. CIN2 in particular is an equivocal precancer that has limited reproducibility and is a heterogeneous mix of infections with carcinogenic potential and those that will regress. Note that we will use the CIN2/3 terminology in reference to past studies in which it was used, but “precancer” when discussing future studies. Regression of precancer through immune recognition and cell-mediated immune control is possible, but less likely than the control of earlier productive infections. CIN2 regression is more common in younger (<25 years) women, non-immunosuppressed populations and those infected with less carcinogenic HPV genotypes i.e., HPV39/51/56/59/68.[7] There are no reliable cross-sectional or short-term predictors of regression. The risk of invasion of a precancer is a function of virus genotype, time and accumulation of genetic changes needed to overcome the cellular safeguards against malignant progression.

As the true underlying natural history of HPV to precancer or cancer cannot be observed due (1) methodological limitations inherent in prospective studies (2) limitations of current measurement assays, and (3) ethical obligations to treat detected precancers, estimates of type-specific transition risks are based on the “average” observable natural history of the different HPV genotypes at the population level. Beyond genotypes, studies have shown that certain variants of HPV16 are associated with higher risks of cancer [8], [9], [10], and that individual responses to some HPV lineages and sub-lineages may differ by race/ethnicity [11].

Evaluation of therapeutic vaccine efficacy will require identifying the relevant transition risk(s) on the causal pathway for: (1) clearance of prevalent HPV16/18 infection, and (2) regression of HPV16/18-associated precancers accompanied by viral clearance. Regarding clearance, there is evidence [12] that the vast majority of HPV infections are cleared within 12–24 months. Infections that persist beyond this period are at a higher risk of precancer [13], [14].

John Doorbar described molecular carcinogenesis and associated cytologic and molecular biomarkers, which may help address these challenges (Supplement Section 6). Nicolas Wentzensen discussed two biomarkers, HPV methylation and p16/Ki-67 dual stain, which may be particularly relevant to therapeutic vaccine trials (here and Supplement Section 7). Methylation of CpG sites in HPV L2 and L1 genes is associated with precancer [15], [16]. The sites vary by HPV type and can indicate the “causal type” underlying CIN3 lesions when multiple infections are present [15], [17], [18], [19], [20]. HPV16 methylation in cytology specimens has been associated with a 50 % risk of underlying prevalent CIN3 [20]. The HPV 16 methylation assay is currently a research assay, however, p16/Ki-67 dual stain, a very specific biomarker for transforming HPV infections, was recently approved by the US FDA for triage of patients with carcinogenic HPV infection [21] based on data showing that a positive dual stain indicates increased risk of precancer warranting colposcopy referral and a negative dual stain test was associated with low cumulative risk of CIN3+ [22]. Clinical recommendations for use of p16/Ki-67 dual stain in management of abnormal screening results were just published [23].

For monitoring during a clinical trial, HPV methylation and p16/Ki-67 dual stain may be useful for (1) defining populations with increased or lower risk of precancer, (2) assessing responses in transforming versus productive infections by study arm, (3) measuring treatment success (decreased detection) without the need for biopsy, or (4) for identifying disease risk (increased detection) requiring clinical action.

### 1.3 Immune responses supporting viral clearance and/or precancer regression

Understanding the immune responses responsible for natural viral clearance and precancer regression, including T-cell response and memory, might aid the development of effective therapeutic vaccines and the identification of appropriate immune monitoring assays for licensure trials. Key questions include whether there is a quantitative relationship between measurable vaccine immunogenicity and efficacy, whether inducing long term T-cell memory is important, and whether the same immune mechanisms are involved in viral clearance versus precancer regression.

Margaret Stanley pointed out that upon HPV infection, there first is an “immune ignorance” phase in which there is viral evasion of innate and adaptive immunity. (Supplement Section 8) Upon dysregulation of E6 and E7 expression, an “immune compromised” phase follows, where there is an immunosuppressive milieu, with tolerogenic cytokines, downregulation of proinflammatory cytokines and chemokines, and dominance of regulatory T-cells [24]. Therapeutic vaccination should provide benefit by overcoming “immune ignorance” in the infection stage. Ideally, therapeutic vaccination should initiate an immune response that (1) clears infection, (2) induces lesion regression, and (3) generates effective immune memory. It should amplify any existing immunity and modulate or reverse the immunosuppressive milieu in the infected microenvironment. To do so, the vaccine should induce an effective cytotoxic anti-viral response by antigen-specific T-cells. Correct initial programming for the vaccine-induced T-cells is crucial for both magnitude and sustainability of effective vaccine-induced protection. Finally, the vaccine should induce development of effective immune memory that is both systemic and local, as local infiltration of effector T-cells at the HPV-affected site plays a critical role in eliminating HPV lesions [25], [26].

Rafi Ahmed highlighted points of consideration for immune responses in the scenario of persistent viral infection (Supplement Section 9). He emphasized that a therapeutic vaccine must overcome the immunosuppressive environment and inhibitory receptors, so selecting adjuvants that increase antigen presenting cell activity will help induce the critical stem-like T-cells that play a central role in the initial programming and shaping the appropriate response. The vaccine platform is also important and must elicit strong antigen specific CD8+ cytotoxic responses. As both systemic and mucosal routes of administration have benefits, a systemic prime with mucosal boost may be ideal.

Connie Trimble summed up her experience in clinical trials of therapeutic HPV vaccines (Supplement Section 10). Therapeutic HPV vaccine trials to date have found that effective vaccination must be followed by immune changes in the HPV-affected mucosa – peripheral immune responses have not correlated with histologic regression. Therapeutic vaccines may need to include strategies to activate the lesional vascular epithelium, to ensure access of the vaccination-induced effector T-cells to the relevant site. Moreover, lesion regression and viral clearance are not synchronous, with lesion regression often occurring first.

In conclusion, therapeutic vaccines were considered most feasible at the stage of persistent infection. Suitable adjuvants and vaccination routes should be used to ensure induction of appropriate T-cells, most likely CD8+, effector responses, and their being able to access the sites of HPV infection. Clinical studies suggest that evaluation of local mucosal immune responses will likely be the most informative.

### 1.4 Design, regulatory, and ethical considerations for therapeutic vaccine trials

Some of the key considerations for therapeutic HPV vaccine trials, depending upon which indication is being sought, include whether durable clearance of HPV infection could be considered sufficient to support initial licensure, what testing methods are sufficient to document primary study success for viral clearance and precancer regression, and what time frame would be acceptable for assessing outcomes. Other open questions include minimal level of efficacy necessary to support licensure considering the potential public health impact, appropriate sample size for assessing viral clearance and lesion regression endpoints and identifying any secondary outcomes that may support licensure and implementation. The expectation for inclusion of women with HIV at some point during the product development was noted as were concerns about potential interactions between HPV clearance (with or without vaccine) and and acquisition of HIV. Ethical considerations for study conduct were reviewed by Claudia Emerson and summarized in the supplemental materials. She focused on the tension between conducting a randomized controlled clinical trial with interpretable results with provision of standard of care therapy that might intervene on study endpoints (Supplement Section 11).

Designing therapeutic HPV vaccine trials with appropriate statistical power benefits from control arm data from prophylactic vaccine trials as well as natural history studies. To help estimate the sample size necessary for an efficacy study, several investigators provided unpublished data from control arms of prophylactic HPV vaccine trials or other clinical trials (Table 2).

**Table 2:** Negative HPV 16 test at 6, 12 and 24 months after single prevalent HPV 16 positive test among unvaccinated women in selected clinical trials. (unpublished data).

Prevalent HPV16 at entry	Age range (years), number at timepoint 0 (N)	6 months % (N)^a^	12 months % (N)^a^	24 months % (N)^a^
Kenya (KEN SHE) [37]	15 to 20 (97)	13 % (87)^b^	53 % (46)	86 % (12)
Malawi, Zimbabwe, Uganda, RSA (ASPIRE) [38]	18 to 45 (104)	16 % (82)^b^	43 % (43)	64 % (14)
India (IARC)	18 to 23 (n/a)	NA	67 % (30)	80 % (30)
Costa Rica (CVT) [39]	18 to 25 (n/a)	29 % (4 1 6)	60 % (4 7 0)	80 % (4 7 7)
Costa Rica (NHS)	18 to ∼ 99 (n/a)	46 % (80)	65 % (1 2 2)	77 % (1 2 6)
China (Innovax) [40]	18 to 26 (97)	40 % (92)	62 % (82)	72 % (79)
China (GSK) [41]	18 to 25 (102)	34 % (96)	57 % (95)	83 % (83)

ASPIRE, Advances in Screening and Prevention of Reproductive Cancers Project; CVT, Costa Rica HPV Vaccine Trial; IARC, International Agency for Research on Cancer; KEN SHE, Single-dose HPV vaccination efficacy among adolescent girls and young women in Kenya.

a N = percentage denominator.

b KEN SHE and ASPIRE use the cumulative incidence method and report N as the number at risk at each time point.

### 1.5 Recommendations for HPV TX vaccine trial design

The meeting broke into groups to evaluate the two primary indication statements. A summary of the design considerations resulting from discussions following the presentations are shown below. Table 3, Table 4 include trial design characteristics for therapeutic vaccine trials supporting licensure for treatment of prevalent HPV16/18 infection and treatment of precancer lesions, respectively. Several areas of concern generated active discussion and require further review (see “additional comments” in Table 3, Table 4).

**Table 3:** Indication 1, Considerations for clinical trial design for treatment of prevalent HPV16/18 infection.

*3a. Trial design*		
	Key design considerations	Additional design considerations
Design	Randomized, placebo controlled	NA
Key Inclusion criteria	Women 25–49 years of age Positive test for HPV16 and/or HPV18	Inclusion of women of older age Repeat (≥1 month) test to confirm HPV16/18 infection before enrolling
Key Exclusion criteria	Cervical precancer (any) Immunosuppression (WLWH require study at appropriate development stage)	Exclude or triage subjects via conventional automated cytology or p16/Ki67 Dual Stain
Primary endpoints	Negative test for HPV16/18	If negative by 6 months, remain negative through 24 months of follow-up Negative test confirmed by 2nd test at least one month later
Secondary endpoints	Lack of progression to precancer Time to first negative HPV test (test at 6, 12, 18 and 24 months) Persistent negative test for HPV16/18	Negative test for HPV16/18 at high risk for progression (DS positive / methylation) Exploratory analyses within risk strata
Sample size	See 3b, 3c	Ensure adequate enrollment in 30–35 years of age range
Length of study	NA	24 months for initial readout with post-licensure commitment to follow longer to support validity of clearance/persistence as a surrogate outcome for progressive disease.
Additional data to be collected in clinical development	Safety and reactogenicity Immunogenicity Treatment of infections predicted to be at highest risk of progression (assessed by DS and/or methylation)	NA

*3b. Sample size estimates for study of treatment of women with HPV16/18 prevalent infection, evaluation at 2 years post vaccination (Primary study objective). Range of clearance probabilities derived from published and unpublished data reported in*Table 2				
Probability of clearance in unvaccinated (at 2 years)	True VE	VE to exclude (LL95%CI)	Sample size (per arm) with 80% power	Sample size (per arm) with 90% power
90%	90%	70%	500	630
90%	90%	60%	280	370
90%	90%	50%	200	260
70%	90%	70%	150	200
70%	90%	60%	90	110
70%	90%	50%	70	80

*3c. Sample size estimates for study of prevention of progression to cervical pre-cancer among women with HPV16 prevalent HPV infection, evaluation at 2 years post vaccination (Secondary study objective). Range of progression probabilities from Costa Rica Vaccine Trial (CVT)*[39]				
Probability of progression to CIN2+ among unvaccinated (at 2 years)	True VE	VE to exclude (LL95%CI)	Sample size (per arm) with 80% power	Sample size (per arm) with 90% power
6.7%	90%	70%	730	950
6.7%	90%	60%	430	550
6.7%	90%	50%	310	410
4.2%	90%	70%	1180	1560
4.2%	90%	60%	680	890
4.2%	90%	50%	490	620

DS, dual stain; HPV, human papilloma virus.

HPV, human papilloma virus; VE, vaccine efficacy; LL95%CI, Lower limit of 95% Confidence interval.

CIN, cervical intraepithelial neoplasia; VE, vaccine efficacy, LL95%CI, Lower limit of 95% Confidence interval.

**Table 4:** Indication 2: Considerations for clinical trial design for treatment of HPV16/18 precancer.

*4a. Trial design*		
	Key design considerations	Additional design considerations
Design	Randomized, placebo controlled	NA
Inclusion criteria	Women 25–49 years of age Positive test for HPV 16 and/or HPV 18 Histologic confirmation of precancer	Extend age range to 21–65 years
Exclusion criteria	Confirmed invasive lesion Large lesion requiring immediate excision Immunosuppression Insufficient colposcopy	No visible squamocolumnar junction
Primary endpoints	Regression of cervical precancer [<CIN2 + ] at 9 months after first vaccine dose Negative test for HPV16/18 (repeated after > 1 month) at 9 months after first dose	Confirmed regression of HPV16/18 lesions (i.e. clear HPV type and lesion related to HPV type)
Secondary endpoints	Time to precancer regression Time to negative test for HPV16/18 Persistent lesion regression Persistent viral disappearance	Exploratory analyses within risk strata
Sample size	See 4b	Consider ensuring adequate numbers in 30–35 age range Vaccine:control ratio up to 3:1, depending on available data re regression rates
Length of study	9 months, follow to 12 months if regression to < CIN2 observed.	Some discussants considered 9 month follow up without treatment problematic
Additional data to be generated in clinical development	Safety and reactogenicity Intermediate biomarkers Regression to histologically normal at 9 months post vaccination Prevention of progression to cervical cancer due to virus types targeted by the vaccine Evidence of cellular immune responses post vaccination Protection against future HPV infections or precancer after clearance/regression of existing pathology	NA

*4b. Sample size estimates for study of treatment of women with HPV16/18 cervical precancer (CIN2/3), evaluation at 36 weeks post-enrollment.*				
Probability of regression among unvaccinated	True VE	VE to exclude (LL95%CI)	Sample size (per arm) with >=80% power	Sample size (per arm) with >=90% power
10%	50%	30%	74	98
10%	50%	15%	28	37
10%	50%	0%	17	21
15%	50%	30%	88	117
15%	50%	15%	34	44
15%	50%	0%	20	26
20%	50%	30%	104	138
20%	50%	15%	40	52
20%	50%	0%	23	30

For the sample size (per arm) in last two columns of table, the actual power was 81-87% and 91-94%, respectively.

CIN, cervical intraepithelial neoplasia; HPV, human papilloma virus.

CIN, cervical intraepithelial neoplasia; HPV, human papilloma virus; VE, vaccine efficacy; LL95%CI, lower limit of 95% confidence interval.

## 2 Discussion

### 2.1 Part 1

Breakout #1 focused on the design of a study to evaluate efficacy of a vaccine to treat prevalent HPV-16/18 infections. Topics considered during the breakout session included (1) criteria for inclusion/exclusion, (2) trial endpoints, (3) trial duration, (4) safety assessment, and (5) sample size/power (Table 3). Key issues discussed are summarized below, with a particular emphasis on areas where agreement was reached and those where additional discussion and/or data were warranted.

Inclusion/Exclusion Criteria: Consistent with recommendations made by previous meetings [27], there was general agreement that trials to evaluate clearance of prevalent HPV-16/18 should target women aged 25–49 years. Given that (1) the likelihood of persistence of prevalent HPV infection is influenced by age and (2) prevalent infections are likely to be identified upon screening initiation, the group considered it desirable to ensure sufficient numbers of women aged 30–35 years are enrolled to permit subgroup analyses targeting this age group. There was a general agreement that a virologic endpoint should require detection and confirmation of HPV-16/18 infection using cervicovaginal samples collected at least 1-month apart and tested using an appropriate type-specific HPV-16/18 detection test. Confirmation of infection with two tests taken at least 1 month apart would limit viral detection in the absence of an established infection (e.g.: deposition), which could lead to attenuated findings. To ensure participant safety in a trial of the expected duration (see section on study duration below), women with evidence of high-grade cervical precancer at screening should be excluded and referred to colpo-biopsy.

Trial Endpoints: Efficacy trials with a cancer outcome are neither practical nor ethical so the discussion focused on two alternative surrogate endpoints: clearance of infection and progression of infection to pre-cancer. There was agreement that both endpoints should be considered although a concern voiced by participants in using the viral clearance endpoint was that unless a product has very high efficacy, it may not be clear whether an effective treatment is only leading to accelerated clearance of infections already destined for clearance. An advantage of using progression to pre-cancer as a target is that it is more proximal to cancer although its use as a primary endpoint would require a larger sample size and extended follow-up for adequate statistical power (see Table 3c). There was agreement among a majority of discussants that initial efficacy trials for licensure could focus on virologic clearance as the primary outcome, with progression to precancer as a secondary outcome. An additional secondary objective discussed during the meeting was time to clearance, in conjunction with markers of infection aggressiveness to ensure that the proportion of infections with a higher risk of progression doesn’t increase over time. There was agreement that no immunological markers currently exist that are known to be associated with HPV infection fate and that this could be explored within an efficacy study.

Measurement of Endpoints: To avoid false negative viral clearance results due to either inadequate sample collection and/or a false negative test, the group recommended two consecutive (>= one month interval) negative samples in order to confirm viral clearance. There was also general agreement that it would be desirable to extend participant follow-up beyond the initial study period (see below) to confirm that efficacy estimates are sustained. For the endpoint of progression to precancer, histological measures of progression to pre-cancer would be ideal. This will require colposcopic evaluation and treatment at the end of the initial follow-up period and the implementation of an expert pathology panel blinded to participant arm assignment.

Study Duration: As shown in Fig. 1, a sizeable majority of prevalent HPV infections clear within 2 years of initial detection. A minority progress to precancer, which typically becomes detectable 2–5 or more years after initial detection of infection. As a consequence, evaluation of viral clearance as an endpoint in HPV therapeutic vaccine trials is feasible within a 2-year study framework whereas robust assessment of a therapeutic vaccine’s ability to prevent progression may require longer evaluations. In addition to the evaluation of clearance, evaluation of the molecular characteristics of infections that clear versus persist, and of infections that progress as secondary/exploratory objectives in the initial study period, would provide valuable insights into whether 2-year viral clearance constitutes an adequate surrogate endpoint and whether clearance in the initial two years reflects the longer-term fate of these infections.

Clinical Management: Virological and cytological testing will be required to define eligibility for randomization. Two alternative cytological screening approaches were discussed to exclude the subset of individuals with evidence of precancer requiring further evaluation and possible treatment and thus ineligible: conventional automated cytology and p16-Ki67 dual stain. As described above (and Supplement Section 7), use of p16-Ki67 staining has the advantage of being highly sensitive for prevalent high-grade precancer but the disadvantage of being non-specific, leading to the exclusion of a larger proportion of potentially eligible individuals. Automated cytology has the advantage of being highly specific but the disadvantage of being less sensitive for prevalent high-grade precancer. Since participation in a therapeutic trial will require follow-up for at least 2 years before final colposcopic evaluation and potential treatment, the safety of such an approach vis-à-vis cancer risk will need to be assessed prior to its use. Annual or semi-annual cytology screening and end-of-study colposcopy and appropriate referral was also suggested.

Exploratory markers for “relevant” viral clearance: When using viral clearance as the primary outcome in HPV vaccine therapeutic trials, an important assumption is that viral clearance is a good surrogate for protection against cervical cancer. However, given that <10 % of individuals infected with HPV-16/18 will progress, it is theoretically possible for a therapeutic HPV vaccine to lead to 90 % clearance of the virus without impacting rates of cervical precancer/cancer. Given that the surrogacy of viral clearance has not been established in the context of a therapeutic vaccine, consideration should be given for initial HPV therapeutic trials that use viral clearance as a primary endpoint to incorporate the following into their trial plan:

1. Evaluation of efficacy against precancer as a secondary objective to closely monitor progression rates among non-cleared viruses
2. Comparison of the characteristics of HPV-16/18 infections at entry and at the time of initial readout of the primary trial outcome.
3. Continued monitoring of participants for several additional years (e.g., up to 5 years total) to confirm that vaccination increases viral clearance and reduces progression to precancer over the life-course of the infection.

With respect to #2 above, p16/Ki67 dual stain and viral methylation patterns have been shown to correlate with risk of progression (see Natural History Section). Therefore, it would be informative to ensure that the percentage of infections that are dual stain positive and/or that have high-risk viral methylation pattern have not increased when comparing women with infection at entry and at the time of initial efficacy readout. The performance of such supportive assays needs to be closely examined if intended for use in a regulatory filing.

Vaccine Safety: Trials that evaluate whether an HPV-16/18 vaccine can treat prevalent infections will exclude HPV16/18 negative women for efficiency. However, one of the potential use-case scenarios could be for broad use in young and mid-adult women irrespective of HPV status. As such, data on the safety of a novel therapeutic vaccine when administered to uninfected women or women infected with only other HPV types will not be available from these efficacy trials. While there is no mechanistic reason to believe that the safety profile of any vaccine will differ when administered to HPV16/18 infected and uninfected women, there was a general consensus that additional safety data would be required beyond that generated through the phase 3 efficacy trials, and that such requirements should be defined in consultation with regulatory bodies. Some attendees felt that licensure could only be achieved in the targeted population in which efficacy was demonstrated and even a separate safety study in an unscreened population would be insufficient to support a “general use” indication.

Sample Size and Power: Sample size and power calculations are provided for a 2-arm randomized trial among women with prevalent HPV-16/18 and no evidence of cervical precancer (HSIL) at entry, with an allocation ratio of 1:1 to vaccination versus placebo and a 2-year follow-up period where the primary endpoint is type-specific clearance of infection and the secondary endpoint is progression to precancer. Table 3b summarizes sample size requirement for the primary objective of viral clearance while Table 3c summarizes power for the secondary objective of progression to precancer. Assumptions regarding probability of viral clearance and progression over the 2-year follow-up period were based on data obtained from trials in Latin America, Africa and Asia, as summarized in Table 2. It is assumed that trials will be implemented at sites with existent or opportunistic HPV-based screening so that appropriate HPV-16/18 infected individuals can be identified without the need to implement de novo screening, which would increase sample size requirements.

With a target efficacy of 90 %, a trial would require between 200 and 630 participants per arm to achieve 80–90 % power assuming spontaneous viral clearance rates of 90 % over two years. We also estimate that given a sample size of 430–950 per arm, the power to detect high (90 %) efficacy against progression to precancer as a secondary objective would range from 80 to 90 % assuming a progression rate in unvaccinated of 6.7 % over two years. Alternative sample sizes could be considered based on other scenarios; see assumptions in tables 3b and 3c.

### 2.2 Part 2

Breakout #2 focused on the design of efficacy studies to support the indication of treatment of cervical precancer for which there has been significantly more product development activity and engagement with regulatory authorities. (Table 4) More experience therefore has accumulated with inclusion/exclusion criteria, clinical endpoints, duration of follow-up and clinical management including both published studies and methods reported from studies on public registries [28], [29], [30], [31]. The focus of the discussion was therefore on learnings from these studies and topics of controversy in their design and conduct.

*Clinical considerations for randomized trials of vaccines targeting cervical precancer* (Mark Einstein)

When designing a therapeutic vaccine trial targeting treatment of cervical precancer, it has been important to define what is considered clinically relevant disease among HPV infected individuals. The ‘gold standard’ for entry into trials has been colposcopically-directed biopsy obtained histology. Such a biopsy should have no suspicion of invasions or microinvasion and must be full thickness through the basement membrane. It should be scored either using the traditional CIN numerical approach or the LAST guidelines [32] and including HSIL are acceptable, with the latter being more subjective thus requiring central pathology review. A standard clinical endpoint of no CIN with a negative viral test has been used for trials. However, in some instances of CIN2/3, patients are co-infected with more than one HPV type that can result in clinical manifestations of precancerous lesions that have low risk of progression. Thus, if the clinical endpoint is a combination of HPV testing and histology, challenges may arise with interpretation of discrepancies. A longer timepoint for endpoint analysis may be required in a scenario where HPV16/18 virus is absent, but LSIL remains. Given the high ubiquity of this scenario in patients with HPV 16/18 and precancer and the cost and burden incurred by such testing, with a lack of standardization an alternative approach to assessing if an LSIL is clinically merely a cytomorphologic manifestation of a non-HR HPV type would be to do a longer interval follow-up.

For a primary endpoint, studies have used confirmed histologic regression to <CIN2+ at 36 weeks after first vaccine dose. Because virologic clearance may lag in time, an additional requirement of negative HPV test at a later timepoint (e.g., 52 weeks) has been used. There has routinely been a placebo arm in these trials to compare to natural regression, and the safety of subjects is critical. As such, a high-quality colposcopy at baseline is essential not only for disease ascertainment, but to assure the lesion(s) in question can be safely followed for a limited interval. Colposcopy should consist of at least one biopsy within each distinct colposcopically-identified lesion and likely endocervical curettage (ECC) [33] to rule out endocervical involvement and also to rule out possible microinvasive or invasive disease at baseline. Large lesions rarely have no endocervical involvement, so limitations on lesion size are often specified in enrollment criteria. Screening biopsies should be no longer than 8 weeks prior to enrollment, and confirmation of no disease may still be needed in the case of this time period being longer than 8 weeks or there is any question of colposcopy or biopsy quality.

Colposcopic size of the lesion is of limited use for monitoring response to intervention. Correlation between colposcopy-directed biopsies and final pathology with loop electrosurgical excision procedure (LEEP)/cone biopsy (LEEP/cone) has not been established in CIN2+, and the ALTS trial and the Biopsy Study showed clear differences between colposcopy impressions and biopsy results [34], [35]. Colposcopy-negative exams have been associated with LEEP results that show multifocal microscopic CIN2+ [36]. LEEP/cone is the gold standard for monitoring response in a trial, as four quadrant biopsies can miss precancer or even cancer. HPV18 is associated with high-grade lesions that are difficult to detect and consequently less commonly found, increasing the risk of invasive cancer.

With regards to designing the study arms for a therapeutic vaccine trial impacting precancers, the differences in regression rates between HPV16 and HPV18 lesions and non-HPV16/18 lesions need to be considered. Control arms from various trials over the past 10 years have shown spontaneous regression rates in HPV16/18 subjects over a 6-month period to be around 10 % with estimates up to 20 % in some instances. There are higher regression rates in non-HPV16/18 infections and CIN2 vs. CIN3, suggesting that regressions rate estimates should be carefully considered depending on population studied. An example of the study sample size that might be considered is shown in Table 4b with 98–138 subjects per group (1:1 randomization) with presumed 50 % efficacy to rule-out with lower limit of 95 % confidence interval of 30 % vaccine efficacy.

Recent studies (e.g., Kawana et al. [29]) have confirmed the spontaneous regression rates of ∼10 % but the relative contribution of CIN2 vs. CIN3 or other population-specific differences in the enrolled population may modify expectations requiring close monitoring by a data monitoring committee to advise on study conduct.

Other practical trial design issues to consider include over-stratified trial designs (may lead to long screening windows due to pathology review requirements), HPV type prescreening, and most importantly, close monitoring for safety during the follow-up period.

## 3 Conclusion

The licensure and use of safe, effective HPV therapeutic vaccines can significantly contribute to reducing the incidence of HPV-related precancer and preventing deaths from cervical cancer, especially in areas with limited screening and treatment options. This report's recommendations for clinical trial design aim to guide the advanced stages of clinical trials and the approval process for these vaccines but also can inform thinking in early product development. There are notable gaps for guidance to developers concerning vaccines intended to treat existing HPV infections. Further research into the natural history of HPV infections, using consistent definitions, statistical methods, and uncertainty estimates, would aid developers in designing appropriate studies. The design approach for studies of vaccines targeting cervical precancers has regulatory precedent and therefore its path is relatively clear, with modest expected sample sizes. For vaccines intended to treat prevalent infection, the licensure path is less clear and will require early and thoughtful engagement with regulators. Particular attention should be paid to excluding the possibility that a vaccine may speed the clearance of virus otherwise destined for spontaneous clearance without affecting those destined to progress. Nonetheless, there is precedent for managing such uncertainties as has been achieved with other vaccine licensure, through regulatory pathways that follow step-wise conditional or accelerated approval while more definitive evidence is generated.

## Funding

This work was funded by the Bill & Melinda Gates Foundation.

## CRediT authorship contribution statement

**Peter M. Dull:** Writing – review & editing, Conceptualization. **Sharon L. Achilles:** Writing – review & editing, Conceptualization. **Rafi Ahmed:** Writing – review & editing, Conceptualization. **Ruanne V. Barnabas:** Writing – review & editing, Conceptualization. **Nicole G. Campos:** Writing – review & editing, Conceptualization. **Keith Chirgwin:** Writing – review & editing, Conceptualization. **Jamie A. Cohen:** Writing – review & editing, Conceptualization. **Silvia de Sanjosé:** Writing – review & editing, Conceptualization. **John Doorbar:** Writing – review & editing, Writing – original draft, Project administration, Conceptualization. **Mark H. Einstein:** Writing – review & editing, Conceptualization. **Claudia I. Emerson:** Writing – review & editing, Conceptualization. **Sami L. Gottlieb:** Writing – review & editing, Conceptualization. **Allan Hildesheim:** Writing – review & editing, Conceptualization. **Youlin Qiao:** Writing – review & editing, Conceptualization. **Paul Ruff:** Writing – review & editing, Conceptualization. **Joshua Sampson:** Writing – review & editing, Conceptualization. **Peter Sasieni:** Writing – review & editing, Conceptualization. **Mark Schiffman:** Writing – review & editing, Conceptualization. **Haina Shin:** Writing – review & editing, Conceptualization. **Margaret A. Stanley:** Writing – review & editing, Conceptualization. **Cornelia L. Trimble:** Writing – review & editing, Conceptualization. **Nicholas Wentzensen:** Writing – review & editing, Conceptualization. **Angelika B. Riemer:** Writing – review & editing, Writing – original draft, Project administration, Conceptualization. **John T. Schiller:** Writing – review & editing, Writing – original draft, Project administration, Conceptualization. **Aimée R. Kreimer:** Writing – review & editing, Writing – original draft, Project administration, Conceptualization.

## Declaration of competing interest

The authors declare that they have no known competing financial interests or personal relationships that could have appeared to influence the work reported in this paper.

## Data Availability

No data was used for the research described in the article.
