# Supplementary material for: Meeting report: Considerations for trial design and endpoints in licensing therapeutic HPV16/18 vaccines to prevent cervical cancer
Source: Vaccine. 2024 Nov 14;42(25):None. doi: 10.1016/j.vaccine.2024.07.001 (PMC11413486; doi:10.1016/j.vaccine.2024.07.001)
Supplement: Supplementary Data 1 [file mmc1.docx]

Meeting report: Considerations for trial design and endpoints in licensing therapeutic HPV16/18 vaccines to prevent cervical cancer (Supplemental content)

September 12-13, 2023, Bill & Melinda Gates Foundation, Washington, D.C

Attendees:

Sharon Achilles, Bill & Melinda Gates Foundation

Rafi Ahmed, Emory Vaccine Center

Mark Alderson, Maxvax

Ruanne Barnabas, Massachusetts General Hospital

Nicole Campos, Harvard University

Keith Chirgwin, consultant

Jamie Cohen, Institute for Disease Modeling, Bill & Melinda Gates Foundation

Silvia de Sanjosé, consultant at the National Cancer Institute & ISGlobal (Spain)

Marta del Pino, Clinic Hospital of Barcelona, University of Barcelona

Joao Dias, BioNTech

John Doorbar, University of Cambridge

Peter Dull, Bill & Melinda Gates Foundation
Mark H. Einstein, Rutgers New Jersey Medical School

Claudia I. Emerson, McMaster University

Doran Fink, Moderna

Sami Gottlieb, World Health Organization

Allan Hildesheim, consultant

David Hokey, Inovio

Karin Jooss, Gritstone Bio

Holger Kanzler, Bill & Melinda Gates Foundation

David Kaslow, Food and Drug Administration

Aimée Kreimer, National Cancer Institute

Annette Lommel, Paul-Erlich-Institut

Chemtai Mungo, University of North Carolina, Chapel Hill

Joel Palefsky, UCSF School of Medicine

Youlin Qiao, Chinese Academy of Medical Sciences & Peking Union Medical College

Angelika Riemer, German Cancer Research Center (DKFZ)

Jeff Roberts, Merck

Paul Ruff, South African Health Products Regulatory Authority, University of Witwatersrand Faculty of Health Sciences

Jerald Sadoff, Janssen R&D

Joshua Sampson, Food and Drug Administration

Peter Sasieni, Queen Mary University of London

Mark Schiffman, National Cancer Institute

John Schiller, National Cancer Institute

Haina Shin, Bill & Melinda Gates Foundation

Margaret Stanley, University of Cambridge

Dereck Tait, Consultant, Barinthus Biotherapeutics

Xin Tong, Walvax

Connie Trimble, Johns Hopkins Medicine International

Sara Vernam, Bill & Melinda Gates Foundation

Nicolas Wentzensen, National Cancer Institute

TC Wu, Johns Hopkins Medicine International

Table of contents

|  |  | Page |
| --- | --- | --- |
| Section 1 | IARC/NCI meeting on primary endpoints for efficacy studies of HPV prophylactic vaccines | 3 |
| Section 2 | WHO preferred product characteristics for HPV therapeutic vaccines | 3 |
| Section 3 | Summary of previous and current therapeutic HPV vaccine products | 4 |
| Section 4 | Potential impact of therapeutic HPV vaccine amidst other interventions | 5 |
| Section 5 | Burden of oncogenic HPV types and disease | 7 |
| Section 6 | Molecular carcinogenesis and tissue markers | 8 |
| Section 7 | Cytologic and molecular biomarkers as intermediate endpoints | 9 |
| Section 8 | Immune responses necessary for viral disappearance | 12 |
| Section 9 | Therapeutic vaccine-induced T cell responses and importance of memory | 13 |
| Section 10 | Systemic immune responses: how and which to monitor? | 13 |
| Section 11 | Ethical considerations for clinical trials in setting of available HPV therapeutics | 14 |
| References |  | 15 |

**Section 1. IARC/NCI meeting on primary endpoints for efficacy studies of HPV prophylactic vaccines - How previous efforts facilitated new product development**

John Schiller (US National Cancer Institute) presented an overview of relevant past HPV prophylactic vaccine development history. In 2013, a committee of experts sponsored by the International Agency for Research on Cancer and the United States (US) National Cancer Institute met to consider primary endpoints for efficacy trials of second-generation prophylactic HPV vaccines.[1,2] The first generation vaccines were licensed based on randomized controlled trials (RCTs) demonstrating prevention of grade 2 and above cervical intraepithelial neoplasia (CIN2+) by vaccine-included HPV types, which required large trials with long follow-up times. Other challenges included reproducibility of histologic endpoints and lesion attribution in the setting of multiple infections with different HPV types.[3] Advances in scientific understanding of HPV-associated cancers and ten years of post-licensure data from the first-generation vaccines led to interest in identifying alternative efficacy endpoints, including virologic or immunologic endpoints, to streamline future vaccine development where possible.

The committee recommended that immunologic non-inferiority was sufficient for licensure of alternate dosing schedules (i.e. two dose schedules for younger adolescents) or for L1-based VLP vaccines similar to licensed vaccines. The committee also agreed that demonstration of protection against 6-month persistent infection should be required for vaccines targeting new HPV types or to validate a dosing schedule (e.g. single dose) with inferior immune response. For new vaccine technologies, if there is consensus that protection is mediated by neutralizing antibodies, then demonstrating protection against 6-month persistent infection should be sufficient for licensure.[1,2]

As a result of these recommendations, studies have since been used to adopt a two-dose schedule in adolescents,[4] extend vaccine indication to anal cancer,[5] to reconfirm immunogenicity against HPV types 6, 11, 16, and 18 in the second generation nonavalent vaccine trial,[6] and to license Serum Institute of India’s HPV4 vaccine in India (Cervavac).[3,7] More recently, this guidance provided a roadmap to study and ultimately support the recommendation of HPV vaccines as a single-dose administration to persons aged 9 to 20 years as articulated by the updated World Health Organization (WHO) Position Paper on HPV vaccines in 2022.[8] There are important differences in the development status of HPV prophylactic versus therapeutic vaccines at the time of these meetings but the impact from the 2013 HPV meeting has been notable for the many regulatory and policy updates that followed.

**Section 2. WHO preferred product characteristics for HPV therapeutic vaccines**

Sharon Achilles (Bill & Melinda Gates Foundation) presented background on the public health need for HPV therapeutic vaccines, and Sami Gottlieb (World Health Organization) presented WHO’s draft preferred product characteristics (PPC) for HPV therapeutic vaccines. WHO has set target goals towards the elimination of cervical cancer to be met by the year 2030, including that 90% of girls worldwide are vaccinated with HPV prophylactic vaccines by age 15, 70% of women are screened with a high-performance test (e.g., HPV DNA testing) by ages 35 and 45, and 90% of women with precancer receive treatment and 90% of women with invasive cervical cancer receive appropriate management.[9] A key challenge to achieving these goals is inequitable access to prophylactic HPV vaccination. An estimated 16% of girls in LMICs have received the first prophylactic vaccination dose and an estimated 12% have received two doses.[10] An even greater challenge may be scaling up cervical cancer screening and treatment. Many countries lack screening and treatment programs, which can be difficult to implement in low resource settings. On average, only around 10% of women in LMICs have ever received cervical cancer screening.[11][ref] Modeling analyses of the impact of the WHO elimination strategy on mortality in 78 LMICs found that prophylactic vaccination of girls would avert an estimated 4.8 million deaths by 2070.[11] Given that effects on cervical cancer are delayed 20-40 years after vaccination, prophylactic vaccination plus one lifetime screening, which has a more immediate impact, was estimated to prevent 13.3 million deaths by 2070, while the full elimination strategy (prophylactic HPV vaccination, screening, and treatment of existing disease) would prevent nearly 15 million deaths.[12]

At the time of the meeting, WHO was finalizing preferred product characteristics (PPCs) for therapeutic HPV vaccines through a global consultative process. PPCs represent WHO’s preferences for potential vaccines (indication, target population, safety and efficacy considerations, etc.) to optimally address the global public health need, providing early guidance on vaccine attributes and data needs for more rapid vaccine development and implementation in LMICs.[13] Two sets of PPCs were developed to illustrate key considerations according to two potential use cases for a therapeutic HPV vaccine. The first (PPC 1) focuses on clearance of infection, at a minimum HPV16/18, and/or prevention of associated high grade precancers in adult women in settings where a high proportion of women have not received a prophylactic vaccine. Because scaling up screening and treatment capacity will be challenging in many places, a therapeutic vaccine could fill this gap by reaching those already infected to reduce precancer and cancer. (Table 1, main text)

A therapeutic vaccine could also offer a simpler, less invasive treatment option in places where screening is available, which could in turn increase the proportion of patients treated. For this use case, PPC 2 focuses on regression of high-grade precancerous lesions, at a minimum those due to HPV 16/18, and the vaccine would be targeted to those with a positive screening test requiring treatment. For this use case, clinical endpoints, including time frame for assessing regression and whether associated viral disappearance is essential, will need to be refined in discussion with regulators.

**Section 3. Summary of previous and current therapeutic HPV vaccine products**

Haina Shin (Bill & Melinda Gates Foundation) presented an overview of the current vaccine products in development. Over the past twenty years, there has been a large number of therapeutic HPV vaccine clinical trials, mostly conducted in North America and Europe, but few of these have reached late phase trials. Most of these products have been targeted towards precancer and cancer. Vaccines against cervical cancer were often administered with additional therapeutics such as checkpoint inhibitors or chemotherapeutics to boost efficacy. For these products, primary endpoints focused on tolerability and adverse events, while secondary endpoints frequently focused on progression-free survival and duration of response. For vaccines that targeted precancerous lesions, defined as CIN2+ or HSIL, disease regression and viral clearance were the most common primary endpoints, and immunogenicity a frequent secondary endpoint. Fewer clinically tested vaccines have targeted high risk HPV infection, but the endpoints for these candidates were often similar to those targeting precancerous lesions. For these products, a negative HPV test and histological regression of low-grade lesions were the most common primary endpoint, and immunogenicity a frequent secondary endpoint. Different vaccine platforms have been used across all candidates, including nucleic acid (mostly DNA-based) delivered by electroporation, viral vector, and protein/peptides. (Figure 2)


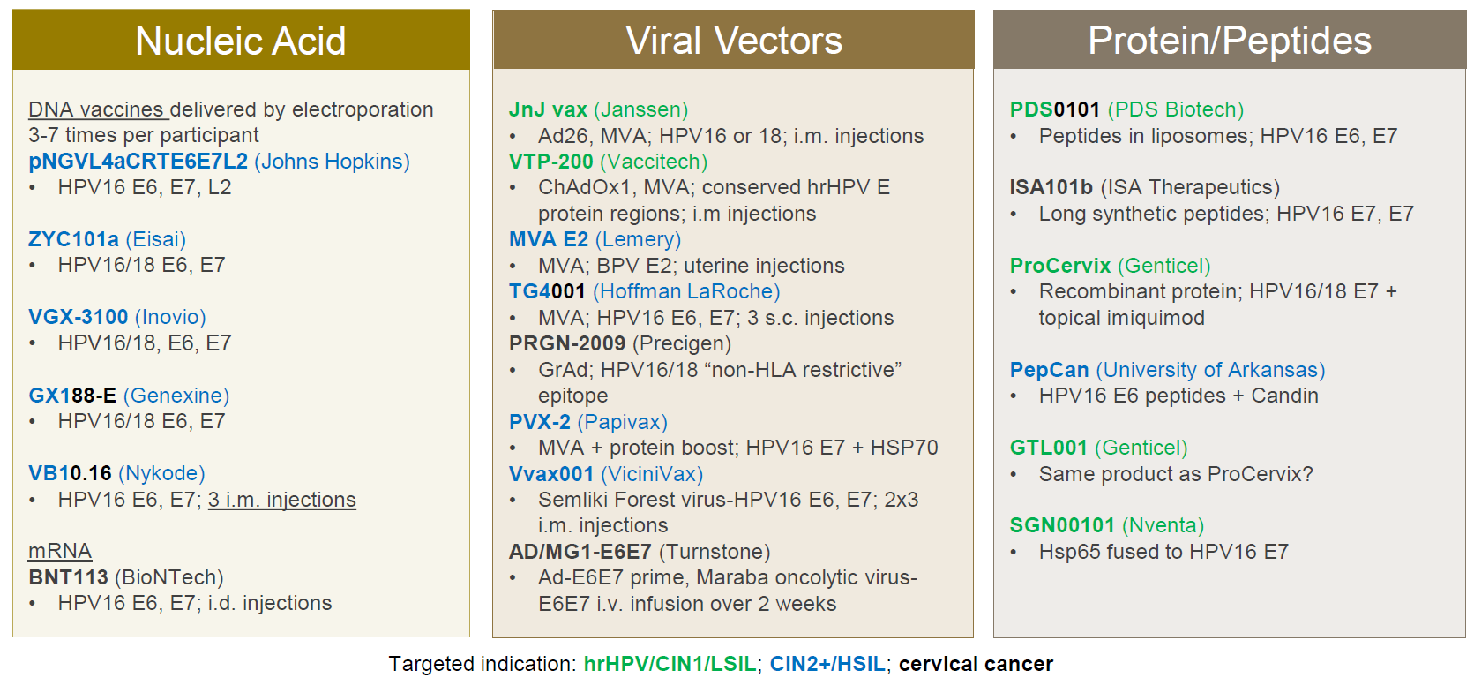


Figure S1 Therapeutic vaccine products that target HPV listed on [clinicaltrials.gov,](https://www.clinicaltrials.gov/) grouped by vaccine platform.

Only one vaccine based on an HPV protein targeting cervical precancer lesions has progressed to a phase 3 study. This vaccine, VGX-3100 (Inovio, Plymouth Meeting, PA) was studied in a pair of late phase studies (REVEAL 1 (ClinicalTrials.gov ID: NCT03185013) and REVEAL 2 (NCT03721978)) and is based on a recombinant plasmid targeting HPV16/18 E6 and E7 proteins and delivered by intramuscular injection followed by electroporation.[14] There are no published results but reported results from the phase 2b trial demonstrated that 49.5% of VGX-3100 recipients versus 30.6% of placebo recipients had histopathological regression (percentage point difference 19.0 [95% confidence interval (CI) 1.4-36.6], p=0·034).[15] The US program was halted although a late development clinical trial has continued with the same product in China. Another large-scale study was conducted for MVA E2 (Virolab/Lemery, Mexico; completed in 2014), a modified vaccinia virus Ankara (MVA) vaccine containing the E2 protein of bovine papillomavirus type1 delivered in multiple doses by uterine injection and designed to elicit localized cytotoxic and innate immune responses not a direct HPV antigen-specific response.[16]

Despite the paucity of phase 3 clinical trials, therapeutic HPV vaccine development continues in preclinical and early phase clinical studies. A shift towards a therapeutic vaccine that can halt disease progression in early stages of HPV pathogenesis and prevent cancer by controlling viral infection will likely require vaccine platforms that can robustly induce mucosal cellular immunity as well consideration of additional HPV antigenic targets.[17]

**Section 4. Potential impact of therapeutic HPV vaccine amidst other interventions**

Jamie Cohen (Institute for Disease Modeling, BMGF) described previous modeling studies by the WHO Cervical Cancer Elimination Modelling Consortium (CCEMC) which have evaluated pathways to cervical cancer elimination with existing interventions.[18] This research suggests that scaling up screening and treatment would expedite elimination of cervical cancer in most LMICs by 11-31 years (compared to increasing prophylactic vaccine coverage alone, which would not address the large population of women who were older when prophylactic vaccination became available).[19] A modeling study assessing the impact of a therapeutic vaccine in Uganda found that a 100% effective therapeutic vaccine against HPV16/18 could prevent 25-30% of lifetime cervical cancer cases irrespective of prophylactic vaccination, but the benefit was reduced if implementation was delayed.[20] Forthcoming work from the WHO CCEMC estimates that a therapeutic vaccine with 90% efficacy against infection and 50% efficacy against CIN2+ would reduce cervical cancer incidence by 14-24% in Sub-Saharan Africa by 2120, even with no prophylactic vaccination or scale-up of screening/treatment, but that the potential impact of a therapeutic vaccine in LMICs would be reduced in the setting of higher prophylactic vaccine coverage and screening/treatment capacity.

New modeling studies to define residual cancer burden with various scale-ups of prophylactic vaccination and screening/treatment supplement these previous findings by also taking into consideration the impact of potential therapeutic vaccine characteristics, or “important impact levers.” (Figure 1) Analyzed vaccine parameters included effectiveness against HPV infection/CIN2+, cross protection, immune memory, age of vaccination, coverage, doses and year of introduction. These models found that significant cancer burden will remain over the next 35 years, even with optimistic scale-up of prophylactic vaccination and screening/treatment capacity, indicating a role for therapeutic vaccines.


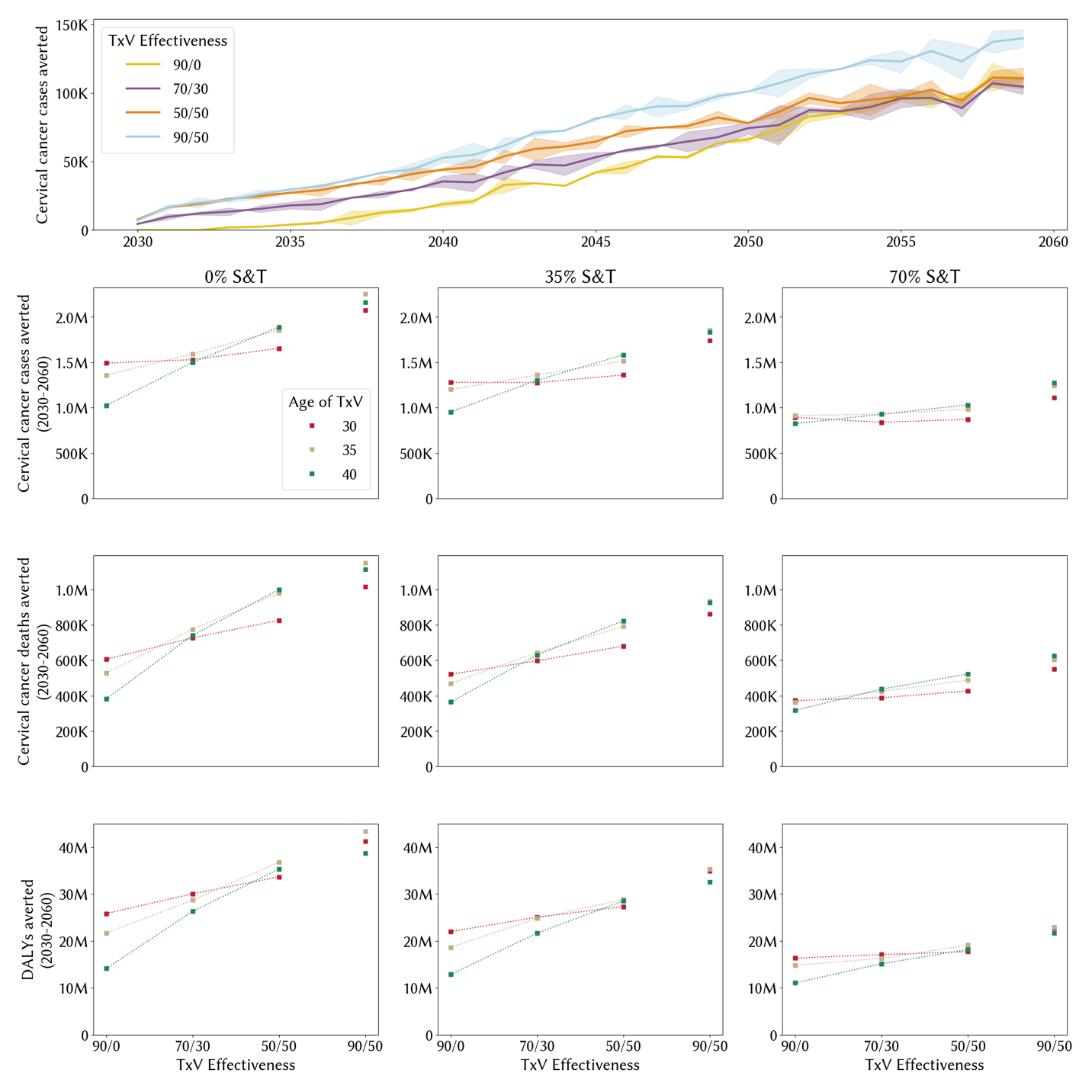


Figure S2. Modeled impact of therapeutic HPV vaccine characteristics on reduction of cervical cancer burden.

Modeling the impact of therapeutic vaccine implementation on cervical cancer burden, with a baseline assumption of 50% coverage and no screening/treatment scale-up, found that as efficacy of the vaccine against CIN2+ increases, older patients see the greatest benefit. Younger patients benefit more from a vaccine that clears infection, with women ages 35-45 receiving the most overall benefit. (Figure S2) Over the short term, modeling found that CIN2+ efficacy was the most impactful vaccine parameter, but durable immune memory plays a large role in preventing cancer 15 years after implementation, suggesting that therapeutic vaccines with action against high-grade lesions that can also induce durable immune memory would have the greatest impact cervical cancer burden reduction.

**Section 5. Burden of oncogenic HPV types and disease**

Silvia de Sanjosé (IS Global) provided an overview of oncogenic HPV types and disease burden. The HPV genome has six early genes: E1 (replication), E2 (replication and transcription), E4 (viral release), E5 (immune evasion), E6 (binds p53 and leads to its degradation) and E7 (binds pRB and leads to its degradation), and 2 late genes: L1 (major capsid protein) and L2 (minor capsid protein).[21] HPV can only complete its full lifecycle through the fully differentiated epithelium. There are around 40 HPV types that can infect the cervix, and risk of viral persistence and progression to cancer varies by type.[22] Thirteen HPV types are known to be oncogenic, including types 16, 18, and 45, as well as the oncogenic groups HPV 31/33/35/52/58 and HPV 39/51/56/59/68. The natural history of HPV infection in the cervix varies and is dependent on age as a proxy for time since sexual debut. The age-specific standardized prevalence of HPV infection also varies by geographic region, with significantly higher prevalence in young people in sub-Saharan Africa. HPV prevalence decreases with age in sub-Saharan Africa, similar to other regions, but remains well above the global average in older age groups. Cultural behavior patterns and screening/treatment programs impact these global variations in HPV prevalence.[23]

HPV16 is universally the most common oncogenic type. Global estimates assembled by the International Agency for Research on Cancer estimate that, among women with normal cytology, HPV prevalence for HPV16 is 2.8%, followed by HPV types 52 (1.5%) 31 (1.2%) and 18 (1.1%).[24] HPV16 prevalence is higher in women with HIV and increases in older age groups, while prevalence decreases with age in HIV negative women.[25] HPV16 also has the highest prevalence (45.1%) in high grade precancer lesions (HSIL/CIN2/CIN3), followed by type 52 (11%), 31 (10.4%), 58 (8.1%), 33 (7.3%) and 18 (6.8%). Infection with more than one HPV type is common in CIN2 (>40%) and CIN3 lesions, though the presence of multiple HPV types is less common in cervical cancer. In cervical cancer, the global prevalence of HPV 16 is 55.2%, with HPV18 having the second highest prevalence (14.2%). HPV types 16 and 18 are the most common types attributed to invasive cervical cancer in all regions of the world, though prevalence of specific non-16/18 oncogenic HPV types varies by region.

Prophylactic HPV vaccines are notably reducing rates of cervical HPV 16/18 infection, precancer, and cancer. Data from Scotland demonstrated a reduction in the cumulative incidence of HPV 16/18 infection from nearly 30% to 14% in a sample of 1000 women over a 3-year period.[26] Similarly, a large cohort study (867,689 women) in Denmark found that women vaccinated at younger ages (<20 years) had significantly lower incidence rates of cervical cancer compared to unvaccinated women during the near 10 year follow-up period.[27] A 2018 meta-analysis of 26 HPV vaccine clinical trials found that vaccination significantly reduced the risk of HPV 16/18 associated CIN2+ (relative risk 0.46; 95%CI: 0.37 to 0.57) in women aged 15-26 years, regardless of baseline HPV infection status.[28] Global prophylactic HPV vaccination coverage is estimated to be around 15%. Once introduced, one dose of vaccine reached 67% of the target population with higher coverage in LMIC (80%).[27] However, two thirds of women ages 30-49 worldwide have never been screened for cervical cancer, with the largest populations of unscreened women in UMICs and LMICs. [11]

**Section 6. Molecular carcinogenesis and tissue markers**

John Doorbar (University of Cambridge) provided an overview of HPV carcinogenesis at the molecular level. Disease outcome after HPV infection depends on both the specific viral gene function and the pattern of gene expression. Following infection, the virus establishes a reservoir of infection in the epithelial basal layers, with productive infection occurring during epithelial differentiation. High risk HPV types can stimulate cell cycle entry in the basal and parabasal layers, driving cell division to allow genome amplification in the upper epithelial layers. The low-risk HPV types stimulate a much lower level of cell cycle entry in the basal layer but stimulate cell cycle entry in the upper epithelial layers to allow genome amplification. Cell cycle markers, such as the cellular MCM protein (indicating cell cycle entry) and the HPV E4 protein (which marks the onset of viral genome amplification) can be used to distinguish between high and low-grade disease in histopathology samples. The pattern of biomarker expression seen in high-risk HPV type infections is heterogenous and reflects the extent of deregulation of the viral E6 and E7 proteins. The biomarker p16 can also be used as a surrogate marker of E6/E7 deregulation. Over time, the deregulated expression of the high-risk HPV E6 and E7 genes (which are often referred to as viral oncogenes), can facilitate the accumulation of secondary genetic errors in the host cell genome. Such lesions are referred to as “transforming infection”, a term which is generally taken as being equivalent to lesions classified as ‘CIN2+’. The E7 and E7 proteins impact many cellular pathways (evasion of growth suppression, DNA damage repair, differentiation, etc.) which can result in the eventual progression to cancer.[29,30]

HPV gene expression deregulation is facilitated at particular epithelial sites, such as the cervical transformation zone between stratified squamous epithelium (covering the ectocervix) and columnar epithelium (covering the endocervix). The cervical transformation zone is a site that can support the normal physiological process of metaplasia, whereby the cervical reserve cells (a specialized type of stem cell that resides under the columnar cells of the transformation zone) can when required, displace the columnar epithelial cells to form a stratified epithelium. It is thought that deregulated viral gene expression occurs more frequently at this site than at ‘conventional’ epithelial sites such as the ectocervix. It is also anticipated that HPV infection occurs more easily at this site, as the cervical reserve cells are more exposed (i.e. closer to the epithelial surface) than the ectocervical basal cells. This explains, at least in part, the vulnerability of the cervical transformation zone to HPV-driven neoplasia and cancer.

HPV immune invasion functions, some of which are encoded by the E6 and E7 genes, play a contributory role in cancer progression by enduring lesion persistence. Indeed, the persistence of disease depends on host factors and HPV type. Current thinking suggests that some individuals will be less able to resolve infection by particular HPV types because of their HLA background. Even in individuals that can generate an effective cell mediated immune response to the virus, the timing of disease clearance may be delayed because of the viruses’ immune evasion functions. The immune response of the host influences both the extent and severity of HPV-associated disease. When “disease-clearance” occurs, this is thought to be as a result of T-cell mediated suppression of viral gene expression rather than through extensive cytotoxic T-cell killing of infected cells.[31] As the HPV genes involved in driving cell proliferation are the same as those involved in evading immune detection, there is a concern that lesions expressing high levels of E6/E7 may clear less efficiently than lower-grade disease. The epithelial niche and host immune response appear to be important regulators of disease outcome. The events that drive natural lesion regression have not yet been well documented with some of our limited understanding coming from the use of animal model systems.


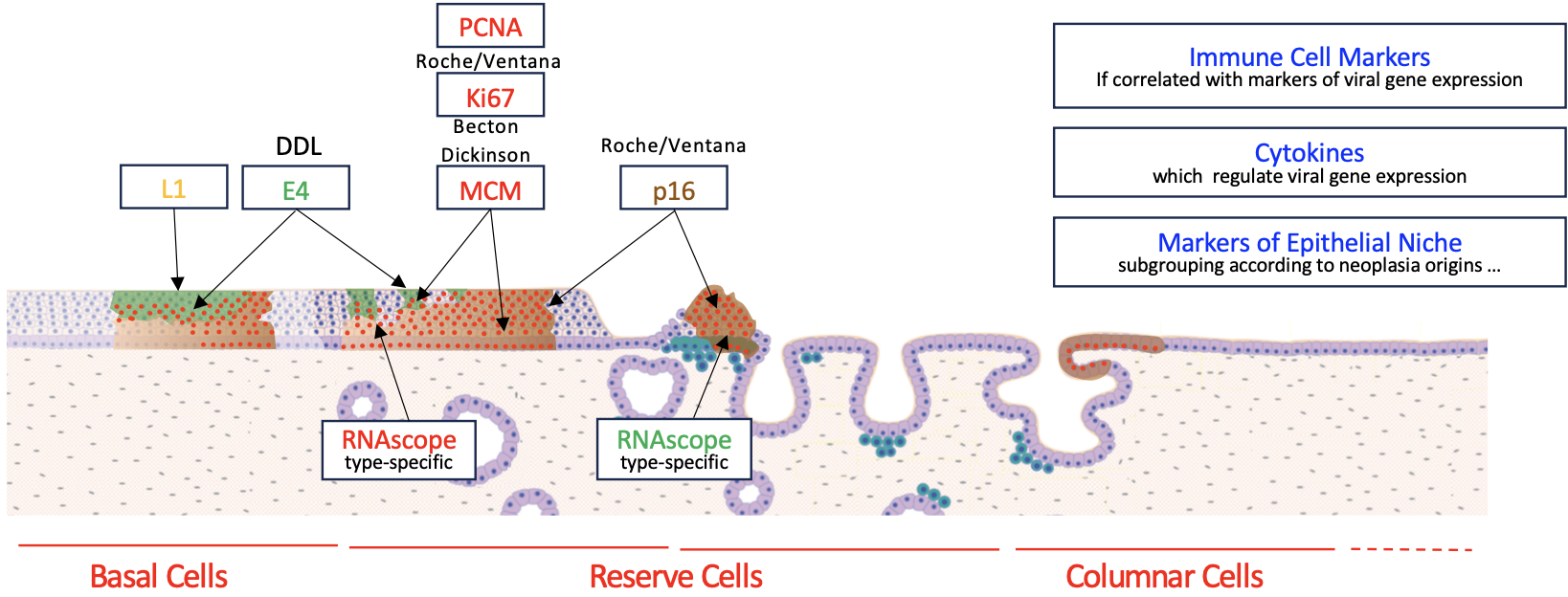


Figure S3. Model of HPV infection with potential biomarkers.

Many of these cellular and immune processes can be detected using markers which allow us to follow the course of disease and to stratify disease severity. These include cytokine markers (which regulate gene expression), markers of epithelial niche, and also immune cell markers. (Figure S3) Immune cell markers in particular, if correlated with markers of viral gene expression, may be useful for monitoring outcomes in therapeutic vaccine trials, and combining biomarkers with spatial information to assess lesions would provide even more information. Spatial mapping approaches such as imprint cytology,[32] although not yet widely utilized, provide us with a non-invasive sampling methodology to allow regular monitoring of disease expansion or regression as part of vaccine evaluation.

**Section 7. Cytologic and molecular biomarkers as intermediate endpoints**

Nicholas Wentzensen (US National Cancer Institute) presented cytologic and molecular biomarkers that are promising as potential intermediate endpoints. Reduction of precancer is an accepted surrogate endpoint for prevention of cervical cancer in prophylactic vaccine trials. However, it can be difficult to monitor precancers over time, due to both methodological issues and safety concerns. Precancers are heterogeneous (lesion size, multiple infections in one lesion, multiple co-occurring lesions with different risk of progression), it is challenging to distinguish between regressing and progressing lesions, cervical biopsy interferes with the natural history of the lesion, and long-term observation of lesions with high risk of progression to cancer without appropriate monitoring may be unethical. Viral infection is also heterogeneous (productive vs. transforming), and reduction of viral detection requires a long observation period to capture the full HPV lifecycle. A recent study performed tissue-based genotyping of precancers to determine the causal HPV type in 690 women with CIN2+ and found that 25% of participants had multiple causal high-risk genotypes, with multiple infections detected within a single biopsy in some cases.[33] This type of approach is critical for understanding the natural history of infections and could be conducted at the end of a vaccine trial but could be impractical for monitoring infections/lesions during a therapeutic vaccine trial.

Cytologic and molecular biomarkers can help address these challenges. Two biomarkers, HPV methylation and p16/Ki-67 dual stain, may be particularly relevant to therapeutic vaccine trials. HPV genomes have 80-120 CpG sites that can be methylated, and particularly methylation of CpG sites on L2 and L1 is associated with precancer.[34,35] Methylation patterns also vary by HPV type and can indicate the “causal type” underlying CIN3 lesions when multiple infections are present. [34,36–39] HPV16, in particular, has strong risk stratification by methylation status: positive HPV16 methylation on cytology specimens has been associated with a 50% risk of underlying prevalent CIN3.[39] The HPV16 methylation assay is currently a research assay but detecting changes in HPV16 methylation after vaccination may be useful as a surrogate of effects on transforming infections and precancers. In addition, detection of methylation during follow-up would indicate a high risk of precancer requiring clinical attention.

The FDA recently approved the p16/Ki-67 dual stain triage test for patients with high-risk HPV infection.[40] The combination of p16, a surrogate marker of HPV-oncogene expression, and Ki-67, a marker of DNA replication that is expressed in cell proliferation, results in a very specific biomarker for transforming HPV infections. In a comparison of screening with partial genotyping and cytologic triage versus dual stain triage, it was found that a negative dual stain test was associated with the lowest cumulative risk of CIN3+.[41] AI-based fully automated detection of dual stain improves accuracy and allows for qualification of dual stain cells on a slide, providing additional thresholds.[42]

For therapeutic vaccine trials, HPV methylation and p16/Ki-67 dual stain may be useful for population selection, either for assessing a subset of transforming/productive infections in the population or for reducing risk of prevalent precancer without the need for colposcopy/biopsy. For example, relevant for the indication of a vaccine treating HPV 16/18 infection, an analysis of data from the Kaiser Permanente trial[41] shows that the HPV16/<HSIL group included 63 participants with CIN3+ prevalent disease, while the HPV16 dual stain negative group had only 6 participants with prevalent CIN3+ disease. (Tables S1a, b) Selecting for a p16/Ki-67 dual stain negative cohort removes subjects at risk for more rapid progression but would also result in fewer cancer endpoints in that group over the 3 year follow up. Using the same study data, when HPV16 infections were stratified by methylation and dual stain, nearly 50% of the methylation positive cases became CIN3+, while of the 67% of the samples that were dual stain positive, around 24% became cases. (Table S2)

Table S1a. Prevalence and incidence of cervical intraepithelial neoplasia (CIN) disease over 3 years in HPV16 infected women aged 25-65 years with/without high-grade squamous intraepithelial lesion (HSIL) and with/without dual stain (DS) positive lesion.

| Total N=1006 | N | CIN2-2/3 prevalent | CIN2-2/3 incident | CIN3+ prevalent | CIN3+ 3-yr incident |
| --- | --- | --- | --- | --- | --- |
| HPV16<HSIL | 836 | 80 | 66 | 63 (7.5%) | 50 (6.0%) |
| HPV16 HSIL | 170 | 48 | 11 | 71 (42%) | 3 (1.7%) |
| HPV16 DS- | 325 | 12 | 20 | 6 (1.8%) | 9 (2.8%) |
| HPV16 DS+ | 681 | 116 | 57 | 128 (18.8%) | 44 (6.5%) |

HPV, human papilloma virus.

Table S1b. Prevalence and incidence of cervical intraepithelial neoplasia (CIN) disease over 3 years in HPV16 infected women aged 25-44 years with/without high-grade squamous intraepithelial lesion (HSIL) and with/without dual stain (DS) positive lesion.

| Total N=790 | N | CIN2-2/3 prevalent | CIN2-2/3 incident | CIN3+ prevalent | CIN3+ 3-yr incident |
| --- | --- | --- | --- | --- | --- |
| HPV16<HSIL | 651 | - | - | 56 (8.6%) | 45 (6.9%) |
| HPV16 HSIL | 139 | - | - | 59 (43%) | 3 (2.2%) |
| HPV16 DS- | 247 | - | - | 4 (1.6%) | 8 (3.2%) |
| HPV16 DS+ | 543 | - | - | 111 (20.4%) | 40 (7.4%) |

HPV, human papilloma virus.

Table S2. Stratification of HPV infections by dual stain and methylation status.

|  | Percent | N | Cases | Risk |
| --- | --- | --- | --- | --- |
| HPV16 meth+^a^ | 31.7 | 1804 | 886 | 49.1 |
| HPV16 meth- | 68.3 | 3895 | 221 | 5.7 |
| All |  | 5699 | 1107 | 19.4 |
| HPV16 DS+^b^ | 67.7 | 681 | 162 | 23.8 |
| HPV16 DS- | 32.3 | 325 | 11 | 3.4 |
| All |  | 1006 | 173 | 17.2 |

^a^Threshold can be modified. ^b^Threshold can be adapted with automated evaluation. DS, dual stain. HPV, human papilloma virus.

DS, dual stain; HPV, human papilloma virus.

For monitoring during a clinical trial, HPV methylation and p16/Ki-67 dual stain may be useful for measuring treatment success (decreased detection) without the need for biopsy, or for identifying disease risk (increased detection) requiring clinical action.

Table S3. Biomarkers as surrogates for precancer detection: Three theoretical scenarios for an observed reduction of HPV infection by 50% in intervention arm

|  | Intervention | Control |
| --- | --- | --- |
| HPV16 | 50% | 100% |
| Methylation positive | 32% | 32% |
| Dual stain positive | 68% | 68% |
| HPV16 | 50% | 100% |
| Methylation positive | >32% | 32% |
| Dual stain positive | >68% | 68% |
| HPV16 | 50% | 100% |
| Methylation positive | <32% | 32% |
| Dual stain positive | <68% | 68% |

HPV, human papilloma virus.

Finally, HPV methylation and p16/Ki-67 dual stain could be used to assess transforming/productive infections by arm. For example, if the vaccine reduces HPV16 infection by 50%, and the proportions of methylation positive and dual stain positive samples do not change between arms, this would indicate that there is equal elimination of productive and transforming infections (though the remaining infections would still need to be monitored). (Table S3) If the proportion of methylation positive and dual stain positive samples increases in the treatment arm, this suggests that vaccination preferentially accelerates clearance of productive infections rather than transforming infections, an undesired scenario. Finally, a decrease in the proportion of biomarker positive cases in the treatment group suggests that the vaccine is preferentially affecting transforming infections, which would be the best-case scenario. As vaccination will not have 100% efficacy against HPV16/18 infection, diligent disease ascertainment in individuals with persistent infections is necessary to rule out precancer, and extended follow-up should be considered to observe progression to precancer.

The conduct of therapeutic HPV vaccine trials is similar to other vaccine studies where a careful assessment of risk-benefit must be made to minimize risk to study participants while generating relevant information to inform regulators. A specific challenge will be balancing acceptable levels of risk in observing pre-cancers against the needed follow-up time for assessing the HPV natural history cycle. Creating a low-risk population based on biomarker assessment minimizes risk but results in fewer cancer outcomes and may not represent the full spectrum of disease due to a higher proportion of transient productive infections. When following precancers, or unselected HPV16-positive participants, intensive disease monitoring is required and observation time of prevalent precancers should be limited.

**Section 8. Immune responses necessary for viral clearance**

Margaret Stanley (University of Cambridge) described the immune responses that are involved in viral clearance. Spontaneous regression of genital warts, which are caused by low-risk HPV types, is accompanied by an intense lymphocytic infiltrate (CD4 lymphocytes in stroma and CD8 in epithelium) and expression of the Th1 type cytokines, suggesting that the mechanism of regression may be Th 1 type cytokine mediated.[43] However, whether this also occurs when CIN1 lesions undergo regression is unknown.


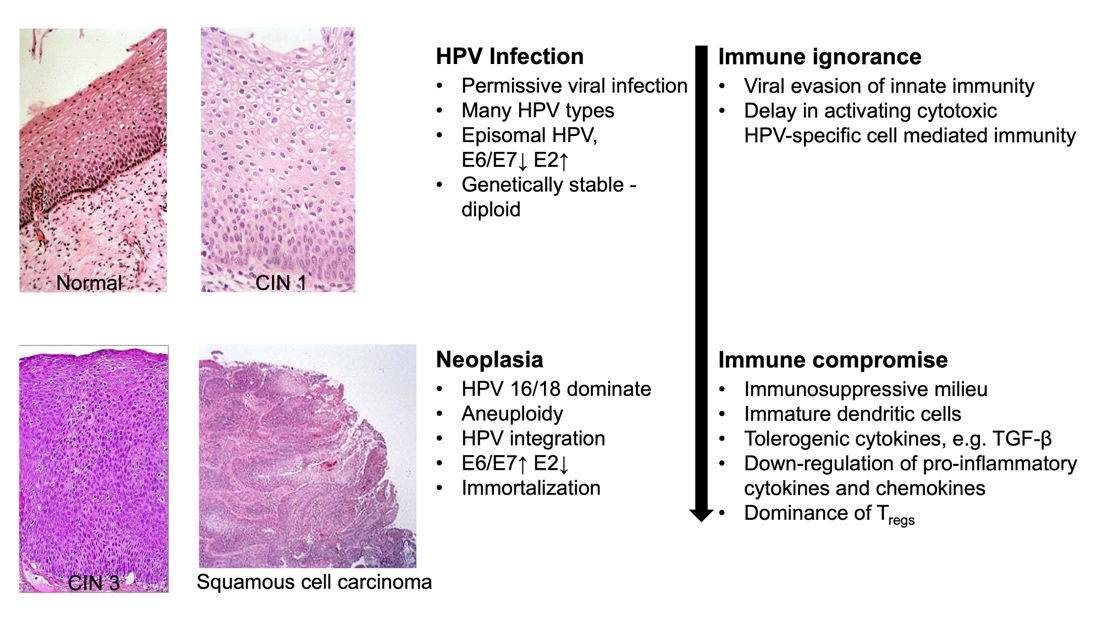


Figure S4. Disease progression in cervical neoplasia.

Disease progression in cervical neoplasia has an “immune ignorance” phase in which there is viral evasion of innate and adaptive immunity. But integration of the viral genome into the genome of the infected cell can eventually lead to loss of E2 expression and dysregulation of E6 and E7 oncogenes, cellular immortalization, and carcinoma. This also results in an “immune compromised” phase, where there is an immunosuppressive milieu, with tolerogenic cytokines, downregulation of proinflammatory cytokines and chemokines, and dominance of regulatory T cells.[21] (Figure S4) A recent study used single-cell RNA-sequencing and spatial transcriptomics data to investigate the spatial distribution of immune cells over the progression from normal cervix to cancer, observing a gradual transition of immune cells from an effective response to dysregulation and exhaustion.[44] In the early infection stage, the naive immune cells do not have antiviral function, but they transition through an antiviral stage during HPV infection. In the precancer stage, there is a balance between antiviral response and immune control, but prevailing immune suppression occurs with invasive cancer. The stage in which there is a balance between antiviral response and immune control is the most critical, and disruption of this balance determines whether precancers regress or progress to cancer.[44,45]

Vaccination should provide benefit by correcting “immune ignorance” in the infection stage. A therapeutic vaccine should initiate an immune response that 1) clears infection, 2) induces lesion regression, and 3) generates effective immune memory. It should amplify any existing effective clearance response and modulate and/or reverse the immunosuppressive milieu in the tumor microenvironment. To do so, the vaccine should induce an effective cytotoxic anti-viral response with antigen-specific T cells imprinted with the ability to kill infected cells. The magnitude of the T cell response is important, and can be optimized through antigen concentration, antigen structure, dosage schedule, and heterologous prime-boost regimen. The induced T cell response should also have breadth, with a diverse repertoire of antigen specific T cells, T cell receptor affinity, and multiple clonotypes specific for the same epitopes, which may offer cross protection. Cytokine polyfunctionality is important, such as co-production of IFN-γ, TNF, and IL-2 by antigen specific CD8+ and CD4+ T cells. Finally, the vaccine should ideally induce development of effective immune memory that is both systemic and local, as local infiltration of effector T cells at the HPV-affected site plays a critical role in eliminating HPV lesions.[46,47]

**Section 9. Therapeutic vaccine-induced T cell responses and importance of memory**

Rafi Ahmed (Emory University) described the vaccine-induced T-cell response. The T-cell response to infection or vaccination goes through several key phases, including rapid expansion (effector phase) in the first month and gradual contraction over the next 6-12 months, during which many cells undergo apoptosis. The remaining differentiated memory CD8+ cells can last for decades, with a very slow contraction.[48] After the acute rapid proliferation phase, if the acute infection is cleared, circulating and tissue-resident functional memory cells persist. But chronic infection can lead to dysfunction in this system resulting in T cell exhaustion: persistent T cells that are no longer able to mount an effector response to the viral antigen.[49,50] During chronic infection, there are three distinct CD8+ T cell states: 1) stem-like CD8+ T (Tpex) cells residing in lymphoid tissues, which respond to the chronic infection by slowly differentiating into 2) circulatory transitory effector cells, which eventually become 3) terminally differentiated CD8+ T cells (exhaustion in the setting of chronic infection).[51–53] The stem-like PD-1+ Tpex cells maintain the T cell response in the setting of persistent antigen and are regulated by TCF-1. They do not express effector molecules but do express the chemokine XCL1 which attracts XCR1+ dendritic cells.

Of these T cell subsets, the Tpex cells residing in lymphoid tissue will likely make up the predominant response to a therapeutic vaccine, by providing the proliferative burst after vaccination and differentiating into transitory effector cells. Tpex cells require both T cell receptor and costimulatory receptor signals for this process. A therapeutic vaccine will need to overcome the immunosuppressive environment and inhibitory receptors, so selecting adjuvants that increase antigen presenting cell activity will be helpful for inducing the Tpex cell response. A 2-dose regimen, with 1 month between doses, and a booster in 6-12 months is likely optimal. For vaccine platforms, nucleic acid and viral vector vaccine platforms may be preferable because the CD8+ response depends on endogenous antigen expression. A carefully designed vaccine may also be effective at preventing subsequent infections from progressing to precancer and cancer, as the Tpex cells will be maintained and will have memory.

**Section 10. Systemic immune responses: how and which to monitor?**

Connie Trimble (Johns Hopkins University School of Medicine) provided an overview of systemic immune responses to HPV infection, precancers and therapeutic vaccines and their significance. Several studies of therapeutic vaccines have provided insight into the nature of effective immune responses, with the most pronounced and relevant changes occurring in the dysplastic mucosa. In a prospective observational study of women with high grade cervical lesions (CIN2+) who were followed for 15 weeks before standard resection, 20% of the HPV16 CIN2+ lesions underwent spontaneous regression during the observation period, with an overall lesion regression rate (all HPV types) of 28%.[54] While peripheral immune responses to HPV were weak and did not correlate with regression,[55] follow-up immunologic analysis of cervical tissue showed that lesion regression could be predicted in part by the ability of T cells to enter the lesional epithelium. Dysregulated expression of vascular adhesion molecules plays an early role in HPV-related immune evasion. One mechanism of immune evasion in CIN3 is via downregulated endothelial expression of the MAdCAM-1adhesion molecule, the ligand for the homing integrin expressed on cervical T cells, α4β7, thereby precluding egress of T cells into the dysplastic epithelium.[56] A therapeutic vaccine may need to include strategies to activate the lesional vascular epithelium.

DNA vaccines have not generated high efficacy in late stage clinical development. In a trial of a DNA vaccine (homologous DNA prime-boost regimen) targeting HPV16 E7 in women with CIN2+, vaccination failed to elicit a local immune response to HPV16 E6/E7 and had no significant clinical effect compared to the observational cohort.[57] Testing with different routes of administration (intradermal, intramuscular, or intralesional) did not improve immunogenicity or clinical response.[58] However, peripheral intramuscular vaccination can induce striking immune changes in the target lesion. In a trial of a heterologous DNA prime-recombinant vaccinia vector-based boost vaccination regimen targeting HPV16/18, the cervical tissue samples obtained 7 weeks following vaccination (week 15) showed a robust lesion-localized effector immune response, despite lack of significant peripheral blood immune response to the vaccine antigen. Post-vaccination, the responding CIN2+ lesions (including those that did not undergo complete regression during the 15-week observation period) had new tertiary lymphoid structures associated with high endothelial venules, lymphocyte access to lesion epithelium, and epithelial cell apoptosis.[59]

**Section 11. Ethical considerations for clinical trials in setting of available HPV therapeutics**

The key ethical considerations for therapeutic HPV vaccine trials were summarized by Claudia Emerson and include equity and access, the public health value of a potential therapeutic vaccine, and the inherent tensions between RCT design and providing standard of care. When considering equity and access, it is critical to acknowledge that women in LMICs bear a disproportionate burden of cervical cancer disease, and the impact of reducing cervical cancer in these populations would impact not only health equity but also socioeconomic development. There is consequently a strong case for conducting studies in LMICs, informed by robust engagement of local stakeholders and in alignment with local social/cultural practices, paying special attention to inclusion of historically vulnerable populations, informed consent, and fair compensation. In addition, there is an increasing presumption towards inclusion of pregnant women in research, unless there are clear scientific or safety reasons to exclude them.

The social value of the research, or the importance of the information that the study is likely to produce, has implications for risk tolerance.[60] In the case of a therapeutic HPV vaccine, the key question centers around whether delaying an established effective treatment for participants is ethical. This can be acceptable if there are compelling scientific reasons for having a placebo, if delaying/withholding the effective intervention will result in no more than a minor increase above minimal risk to participants, and if those risks are minimized with effective mitigation procedures. In the setting where there is limited or no access to effective treatments, justification for delaying treatment requires that the research is aimed towards developing interventions that will be implemented in the population studied and that the trial does not require participants to forgo treatment that they would otherwise receive.

Thinking ahead towards delivery and adoption is also important when designing ethical trials, to ensure that the vaccine product is affordable and accessible and overcomes challenges with uptake in the populations in which it was studied. Of the two indications explored in this report, viral clearance seems easiest to justify ethically, as it has sufficient social value, presents low risk to trial participants, and thus holds potential for boosting confidence in the local community. Clinical trials assessing precancer regression should align with standards of care as there are established protocols for treatment.

References

[1] Lowy DR, Herrero R, Hildesheim A, Participants in the IARC/NCI workshop on Primary Endpoints for Prophylactic HPV Vaccine Trials. Primary endpoints for future prophylactic human papillomavirus vaccine trials: towards infection and immunobridging. Lancet Oncol 2015;16:e226-33.

[2] IARC HPV Working Group. Primary End-points for Prophylactic HPV Vaccine Trials. Lyon (FR): International Agency for Research on Cancer; n.d.

[3] Schiller JT, Castellsagué X, Garland SM. A review of clinical trials of human papillomavirus prophylactic vaccines. Vaccine 2012;30 Suppl 5:F123-38.

[4] Dobson SRM, McNeil S, Dionne M, Dawar M, Ogilvie G, Krajden M, et al. Immunogenicity of 2 doses of HPV vaccine in younger adolescents vs 3 doses in young women: a randomized clinical trial. JAMA 2013;309:1793–802.

[5] Mensah FA, Mehta MR, Lewis JS Jr, Lockhart AC. The Human Papillomavirus Vaccine: Current Perspective and Future Role in Prevention and Treatment of Anal Intraepithelial Neoplasia and Anal Cancer. Oncologist 2016;21:453–60.

[6] Joura EA, Giuliano AR, Iversen O-E, Bouchard C, Mao C, Mehlsen J, et al. A 9-valent HPV vaccine against infection and intraepithelial neoplasia in women. N Engl J Med 2015;372:711–23.

[7] Sankaranarayanan R, Prabhu PR, Pawlita M, Gheit T, Bhatla N, Muwonge R, et al. Immunogenicity and HPV infection after one, two, and three doses of quadrivalent HPV vaccine in girls in India: a multicentre prospective cohort study. Lancet Oncol 2016;17:67–77.

[8] Human papillomavirus vaccines: WHO position paper. vol. 50. World Health Organization; 2022.

[9] World Health Organization (WHO. Global strategy to accelerate the elimination of cervical cancer as a public health problem. Geneva: WHO; 2020.

[10] Bruni L, Saura-Lázaro A, Montoliu A, Brotons M, Alemany L, Diallo MS, et al. HPV vaccination introduction worldwide and WHO and UNICEF estimates of national HPV immunization coverage 2010–2019. Prev Med 2021;144:106399.

[11] Bruni L, Serrano B, Roura E, Alemany L, Cowan M, Herrero R, et al. Cervical cancer screening programmes and age-specific coverage estimates for 202 countries and territories worldwide: a review and synthetic analysis. Lancet Glob Health 2022;10:e1115–27.

[12] Canfell K, Kim JJ, Brisson M, Keane A, Simms KT, Caruana M, et al. Mortality impact of achieving WHO cervical cancer elimination targets: a comparative modelling analysis in 78 low-income and lower-middle-income countries. Lancet 2020;395:591–603.

[13] World Health Organization. WHO preferred product characteristics for therapeutic HPV vaccines. n.d.

[14] Diehl MC, Lee JC, Daniels SE, Tebas P, Khan AS, Giffear M, et al. Tolerability of intramuscular and intradermal delivery by CELLECTRA® adaptive constant current electroporation device in healthy volunteers. Hum Vaccin Immunother 2013;9:2246–52.

[15] Trimble CL, Morrow MP, Kraynyak KA, Shen X, Dallas M, Yan J, et al. Safety, efficacy, and immunogenicity of VGX-3100, a therapeutic synthetic DNA vaccine targeting human papillomavirus 16 and 18 E6 and E7 proteins for cervical intraepithelial neoplasia 2/3: a randomised, double-blind, placebo-controlled phase 2b trial. Lancet 2015;386:2078–88.

[16] Rosales R, López-Contreras M, Rosales C, Magallanes-Molina J-R, Gonzalez-Vergara R, Arroyo-Cazarez JM, et al. Regression of human papillomavirus intraepithelial lesions is induced by MVA E2 therapeutic vaccine. Hum Gene Ther 2014;25:1035–49.

[17] Mo Y, Ma J, Zhang H, Shen J, Chen J, Hong J, et al. Prophylactic and Therapeutic HPV Vaccines: Current Scenario and Perspectives. Front Cell Infect Microbiol 2022;12:909223.

[18] Cohen JA, Stuart RM, Lee S, Klein DJ, Kerr CC, Rao DW, et al. Understanding the key determinants of an HPV therapeutic vaccine: a modeling analysis. MedRxiv 2023:2023.12.04.23299403. https://doi.org/10.1101/2023.12.04.23299403.

[19] Brisson M, Kim JJ, Canfell K, Drolet M, Gingras G, Burger EA, et al. Impact of HPV vaccination and cervical screening on cervical cancer elimination: a comparative modelling analysis in 78 low-income and lower-middle-income countries. Lancet 2020;395:575–90.

[20] Spencer JC, Campos NG, Burger EA, Sy S, Kim JJ. Potential effectiveness of a therapeutic HPV intervention campaign in Uganda. Int J Cancer 2022;150:847–55.

[21] Stanley MA, Pett MR, Coleman N. HPV: from infection to cancer. Biochem Soc Trans 2007;35:1456–60.

[22] Schiffman M, Herrero R, Desalle R, Hildesheim A, Wacholder S, Rodriguez AC, et al. The carcinogenicity of human papillomavirus types reflects viral evolution. Virology 2005;337:76–84.

[23] Schiffman M, Doorbar J, Wentzensen N, de Sanjosé S, Fakhry C, Monk BJ, et al. Carcinogenic human papillomavirus infection. Nat Rev Dis Primers 2016;2:16086.

[24] HPV INFORMATION CENTRE n.d. https://hpvcentre.net/index.php (accessed October 7, 2023).

[25] Wei F, Alberts CJ, Albuquerque A, Clifford GM. Impact of human papillomavirus vaccine against anal human papillomavirus infection, anal intraepithelial neoplasia, and recurrence of anal intraepithelial neoplasia: a systematic review and meta-analysis. J Infect Dis 2023. https://doi.org/10.1093/infdis/jiad183.

[26] Kavanagh K, Pollock KGJ, Potts A, Love J, Cuschieri K, Cubie H, et al. Introduction and sustained high coverage of the HPV bivalent vaccine leads to a reduction in prevalence of HPV 16/18 and closely related HPV types. Br J Cancer 2014;110:2804–11.

[27] Kjaer SK, Dehlendorff C, Belmonte F, Baandrup L. Real-World Effectiveness of Human Papillomavirus Vaccination Against Cervical Cancer. J Natl Cancer Inst 2021;113:1329–35.

[28] Arbyn M, Xu L, Simoens C, Martin-Hirsch PP. Prophylactic vaccination against human papillomaviruses to prevent cervical cancer and its precursors. Cochrane Database Syst Rev 2018;5:CD009069.

[29] Doorbar J, Egawa N, Griffin H, Kranjec C, Murakami I. Human papillomavirus molecular biology and disease association. Rev Med Virol 2015;25 Suppl 1:2–23.

[30] Pal A, Kundu R. Human Papillomavirus E6 and E7: The Cervical Cancer Hallmarks and Targets for Therapy. Front Microbiol 2019;10:3116.

[31] Doorbar J. Host control of human papillomavirus infection and disease. Best Pract Res Clin Obstet Gynaecol 2018;47:27–41.

[32] Shiraz A, Egawa N, Pelt DM, Crawford R, Nicholas AK, Romashova V, et al. Cervical cell lift: A novel triage method for the spatial mapping and grading of precancerous cervical lesions. EBioMedicine 2022;82:104157.

[33] Venetianer R, Clarke MA, van der Marel J, Tota J, Schiffman M, Dunn ST, et al. Identification of HPV genotypes causing cervical precancer using tissue-based genotyping. Int J Cancer 2020;146:2836–44.

[34] Wentzensen N, Sun C, Ghosh A, Kinney W, Mirabello L, Wacholder S, et al. Methylation of HPV18, HPV31, and HPV45 genomes and cervical intraepithelial neoplasia grade 3. J Natl Cancer Inst 2012;104:1738–49.

[35] Clarke MA, Wentzensen N, Mirabello L, Ghosh A, Wacholder S, Harari A, et al. Human papillomavirus DNA methylation as a potential biomarker for cervical cancer. Cancer Epidemiol Biomarkers Prev 2012;21:2125–37.

[36] Mirabello L, Schiffman M, Ghosh A, Rodriguez AC, Vasiljevic N, Wentzensen N, et al. Elevated methylation of HPV16 DNA is associated with the development of high grade cervical intraepithelial neoplasia. Int J Cancer 2013;132:1412–22.

[37] Brentnall AR, Vasiljevic N, Scibior-Bentkowska D, Cadman L, Austin J, Cuzick J, et al. HPV33 DNA methylation measurement improves cervical pre-cancer risk estimation of an HPV16, HPV18, HPV31 and \textit{EPB41L3} methylation classifier. Cancer Biomark 2015;15:669–75.

[38] Lorincz AT, Brentnall AR, Scibior-Bentkowska D, Reuter C, Banwait R, Cadman L, et al. Validation of a DNA methylation HPV triage classifier in a screening sample. Int J Cancer 2016;138:2745–51.

[39] Clarke MA, Gradissimo A, Schiffman M, Lam J, Sollecito CC, Fetterman B, et al. Human Papillomavirus DNA Methylation as a Biomarker for Cervical Precancer: Consistency across 12 Genotypes and Potential Impact on Management of HPV-Positive Women. Clin Cancer Res 2018;24:2194–202.

[40] Wentzensen N, Fetterman B, Castle PE, Schiffman M, Wood SN, Stiemerling E, et al. p16/Ki-67 Dual Stain Cytology for Detection of Cervical Precancer in HPV-Positive Women. J Natl Cancer Inst 2015;107:djv257.

[41] Wentzensen N, Clarke MA, Bremer R, Poitras N, Tokugawa D, Goldhoff PE, et al. Clinical Evaluation of Human Papillomavirus Screening With p16/Ki-67 Dual Stain Triage in a Large Organized Cervical Cancer Screening Program. JAMA Intern Med 2019;179:881–8.

[42] Wentzensen N, Lahrmann B, Clarke MA, Kinney W, Tokugawa D, Poitras N, et al. Accuracy and Efficiency of Deep-Learning-Based Automation of Dual Stain Cytology in Cervical Cancer Screening. J Natl Cancer Inst 2021;113:72–9.

[43] Ball SLR, Winder DM, Vaughan K, Hanna N, Levy J, Sterling JC, et al. Analyses of human papillomavirus genotypes and viral loads in anogenital warts. J Med Virol 2011;83:1345–50.

[44] Guo C, Qu X, Tang X, Song Y, Wang J, Hua K, et al. Spatiotemporally deciphering the mysterious mechanism of persistent HPV-induced malignant transition and immune remodelling from HPV-infected normal cervix, precancer to cervical cancer: Integrating single-cell RNA-sequencing and spatial transcriptome. Clin Transl Med 2023;13:e1219.

[45] Li C, Hua K. Dissecting the Single-Cell Transcriptome Network of Immune Environment Underlying Cervical Premalignant Lesion, Cervical Cancer and Metastatic Lymph Nodes. Front Immunol 2022;13:897366.

[46] Shibata T, Shah S, Evans T, Coleman H, Lieblong BJ, Spencer HJ, et al. Expansion of Human Papillomavirus-Specific T Cells in Periphery and Cervix in a Therapeutic Vaccine Recipient Whose Cervical High-Grade Squamous Intraepithelial Lesion Regressed. Front Immunol 2021;12:645299.

[47] Humeau L, Trimble C, Morrow M, Shen X, Dallas M, Weiner D, et al. DNA vaccine VGX-3100 with electroporation induces regression of cervical intraepithelial neoplasia 2/3 and clears HPV infection with robust T cell responses: results of a randomized, double-blind, placebo-controlled Phase II trial. Journal for ImmunoTherapy of Cancer 2014;2:1–1.

[48] Akondy RS, Monson ND, Miller JD, Edupuganti S, Teuwen D, Wu H, et al. The yellow fever virus vaccine induces a broad and polyfunctional human memory CD8+ T cell response. J Immunol 2009;183:7919–30.

[49] Zajac AJ, Blattman JN, Murali-Krishna K, Sourdive DJ, Suresh M, Altman JD, et al. Viral immune evasion due to persistence of activated T cells without effector function. J Exp Med 1998;188:2205–13.

[50] Murali-Krishna K, Altman JD, Suresh M, Sourdive DJ, Zajac AJ, Miller JD, et al. Counting antigen-specific CD8 T cells: a reevaluation of bystander activation during viral infection. Immunity 1998;8:177–87.

[51] Hudson WH, Gensheimer J, Hashimoto M, Wieland A, Valanparambil RM, Li P, et al. Proliferating Transitory T Cells with an Effector-like Transcriptional Signature Emerge from PD-1+ Stem-like CD8+ T Cells during Chronic Infection. Immunity 2019;51:1043-1058.e4.

[52] Im SJ, Hashimoto M, Gerner MY, Lee J, Kissick HT, Burger MC, et al. Defining CD8+ T cells that provide the proliferative burst after PD-1 therapy. Nature 2016;537:417–21.

[53] Im SJ, Konieczny BT, Hudson WH, Masopust D, Ahmed R. PD-1+ stemlike CD8 T cells are resident in lymphoid tissues during persistent LCMV infection. Proc Natl Acad Sci U S A 2020;117:4292–9.

[54] Trimble CL, Piantadosi S, Gravitt P, Ronnett B, Pizer E, Elko A, et al. Spontaneous regression of high-grade cervical dysplasia: effects of human papillomavirus type and HLA phenotype. Clin Cancer Res 2005;11:4717–23.

[55] Trimble CL, Peng S, Thoburn C, Kos F, Wu TC. Naturally occurring systemic immune responses to HPV antigens do not predict regression of CIN2/3. Cancer Immunol Immunother 2010;59:799–803.

[56] Trimble CL, Clark RA, Thoburn C, Hanson NC, Tassello J, Frosina D, et al. Human papillomavirus 16-associated cervical intraepithelial neoplasia in humans excludes CD8 T cells from dysplastic epithelium. J Immunol 2010;185:7107–14.

[57] Trimble CL, Peng S, Kos F, Gravitt P, Viscidi R, Sugar E, et al. A phase I trial of a human papillomavirus DNA vaccine for HPV16+ cervical intraepithelial neoplasia 2/3. Clin Cancer Res 2009;15:361–7.

[58] Alvarez RD, Huh WK, Bae S, Lamb LS Jr, Conner MG, Boyer J, et al. A pilot study of pNGVL4a-CRT/E7(detox) for the treatment of patients with HPV16+ cervical intraepithelial neoplasia 2/3 (CIN2/3). Gynecol Oncol 2016;140:245–52.

[59] Maldonado L, Teague JE, Morrow MP, Jotova I, Wu TC, Wang C, et al. Intramuscular therapeutic vaccination targeting HPV16 induces T cell responses that localize in mucosal lesions. Sci Transl Med 2014;6:221ra13.

[60] Council for International Organizations of Medical Sciences (CIOMS). International Ethical Guidelines for Health-related Research Involving Humans. CIOMS; 2017.
